# Encapsulation-integrated microneedle platforms for alopecia treatment: a review of controlled drug delivery, immunomodulation, and hair follicle regeneration

**DOI:** 10.3389/fimmu.2026.1952774

**Published:** 2026-10-14

**Authors:** Kerlin E. Balza Boniche, Maria J. Rey-Sanchez, Andres F. Roca-Arroyo, Jhonatan A. Gutierrez-Rivera, Diego Balda, Logan D. Morton, David A. Castilla-Casadiego

**Affiliations:** 1 Department of Biomedical Engineering, University of Miami, Coral Gables, FL, United States; 2 Department of Biomedical Engineering, Tufts University, Medford, MA, United States; 3 Department of Chemical, Environmental and Materials Engineering, University of Miami, Coral Gables, FL, United States

**Keywords:** alopecia, controlled drug release, drug encapsulation, follicular microenvironment, hair follicle regeneration, immunomodulation, microneedles

## Abstract

Alopecia is a multifactorial disorder characterized by progressive hair loss, follicular dysfunction, and, in some cases, immune-mediated damage, significantly affecting patient quality of life. Although conventional therapies such as topical minoxidil, corticosteroids, and oral immunomodulators remain widely used, their clinical efficacy is often limited by poor skin penetration, short therapeutic duration, systemic adverse effects, and the need for frequent administration. In recent years, microneedle-based platforms have emerged as promising minimally invasive systems capable of enhancing transdermal delivery directly to the follicular microenvironment. This review explores the emerging design space of microneedle platforms integrated with advanced encapsulation strategies for alopecia treatment, with particular emphasis on their roles in immunomodulation and tissue regeneration. By examining the interplay between material composition, carrier architecture, and release behavior, we discuss how these systems improve localized delivery while enabling sustained and controlled therapeutic exposure within the hair follicle niche. Recent advances in nanoparticles, nanoliposomes, porous materials, and microsphere-integrated microneedles, as well as light-guiding systems, have expanded the capabilities of conventional polymeric microneedles beyond passive drug delivery. These platforms enhance drug stability, regulate release kinetics, improve follicular targeting, and actively modulate key biological processes associated with alopecia pathogenesis and regeneration. Reported therapeutic effects include suppression of inflammatory signaling pathways, regulation of immune-cell infiltration, stimulation of angiogenesis, reduction of oxidative stress, activation of follicular stem-cell populations, and promotion of the telogen-to-anagen transition. Collectively, these advances position encapsulation-integrated microneedle systems as a promising class of next-generation therapeutic platforms capable of combining localized delivery with biological microenvironment modulation. Finally, this review discusses current translational challenges, including manufacturing scalability, formulation stability, long-term safety, and disease-specific platform design, while highlighting future opportunities for personalized and regenerative alopecia therapies.

## Graphical Abstract

**Figure:**
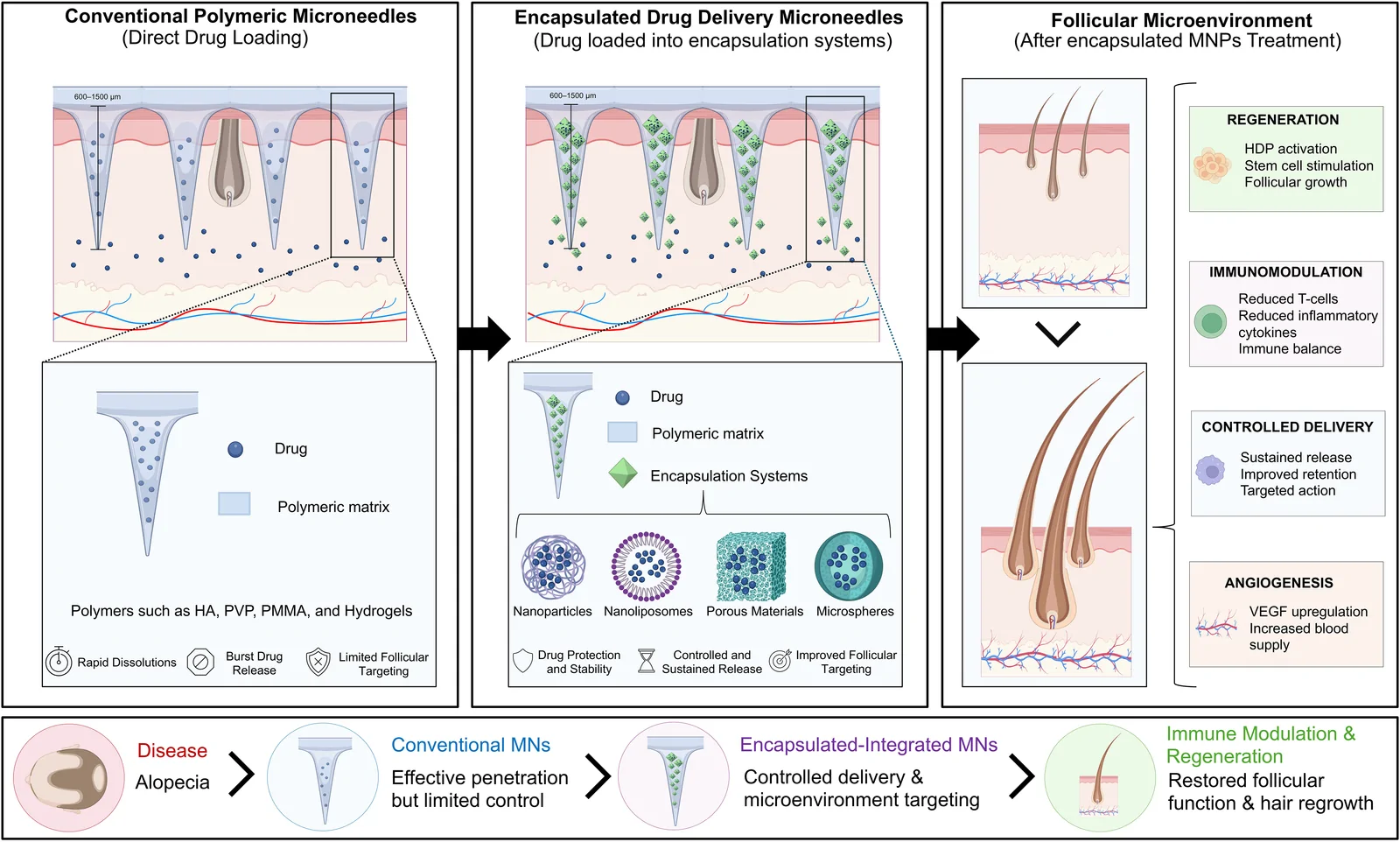

## 1 Introduction

Alopecia encompasses a group of disorders characterized by hair loss, with alopecia areata (AA) and androgenetic alopecia (AGA) representing the most prevalent clinical forms and the primary conditions addressed in this review (1, 2). AA affects approximately 2% of the global population, corresponding to over 150 million individuals worldwide (3), while AGA affects up to 70% of men and 40% of women (4). Although these conditions differ in their underlying pathophysiology, both AA and AGA are influenced by a combination of genetic background, hormonal status, and environmental factors such as psychological stress that contribute to follicular dysfunction and disease progression (1).

Importantly, growing evidence indicates that alterations in the follicular microenvironment play a central role in disease progression (1, 5). In AA, hair loss is primarily driven by immune dysregulation characterized by the collapse of hair follicle immune privilege, which promotes the infiltration of autoreactive CD8^+^ T cells, natural killer cells, and pro-inflammatory cytokines that target follicular structures and disrupt normal hair cycling (1, 6). In contrast, AGA is predominantly associated with androgen-mediated follicular miniaturization; however, chronic low-grade inflammation, oxidative stress, and alterations in the follicular microenvironment have increasingly been recognized as important contributors to disease progression and impaired follicular function (6, 7).

The clinical impact of alopecia extends far beyond physical appearance (8). Hair loss is strongly associated with psychological distress, including reduced self-esteem, anxiety, and depressive symptoms (1, 8). In addition, individuals with alopecia frequently experience emotional burden, social withdrawal, and decreased confidence (8, 9). Alopecia is driven by complex biological mechanisms involving immune dysregulation, inflammation, and alterations in follicular homeostasis that ultimately disrupt normal hair growth and regeneration (3, 5, 8).

Current management includes several therapeutic approaches, including injectable therapies, topical agents such as minoxidil (MXD) and betamethasone, and oral tablets such as finasteride or baricitinib (9). Despite their widespread clinical use, each approach has important limitations. Injectable treatments are commonly administered in AA every 4–6 weeks, depending on clinical response. This can lead to a reduction in patient compliance, as their invasive nature may cause discomfort or pain (10).

Topical therapies such as MXD or corticosteroids, typically applied once or twice daily, are limited by poor penetration through the stratum corneum, leading to suboptimal delivery to hair follicles and reduced therapeutic efficiency (7, 9, 11). Oral therapies, including finasteride, dutasteride, and JAK inhibitors, bypass the skin barrier but are associated with systemic exposure, long-term daily administration, and potential adverse effects that may limit long-term compliance (7, 9).

Microneedles have emerged as promising minimally invasive platforms for alopecia treatment due to their ability to bypass the stratum corneum and enhance localized drug delivery directly to the follicular microenvironment (4, 5, 12). Typically ranging from approximately 600 to 1500 μm in length, depending on the application and target tissue depth, microneedles create transient microchannels in the skin that facilitate transdermal transport while avoiding stimulation of deeper dermal nerves and blood vessels, thereby minimizing pain and tissue damage compared with conventional injection treatments (12, 13). Beyond improving transdermal penetration, microneedles may also contribute directly to follicular regeneration through controlled microinjury and modulation of the perifollicular microenvironment (9, 13).

Traditionally, dissolving microneedles (**Figure 1A**) for alopecia applications are fabricated using biocompatible polymers in which therapeutic agents, including MXD, triamcinolone acetonide (TA), dutasteride (DUT), and tofacitinib (TFB), are incorporated within the polymeric matrix during manufacturing (9, 13). In many conventional formulations, the drug is directly mixed with the polymer solution before micromolding, using polymer-to-drug ratios optimized to preserve needle mechanical integrity while enabling adequate loading and dissolution behavior (14, 15). However, despite their advantages, these simple matrix-loading strategies frequently result in rapid and uncontrolled burst release following microneedle dissolution, which can lead to premature drug depletion, short therapeutic duration, fluctuating local drug concentrations, and the need for repeated administration to maintain efficacy (9, 16). Such limitations are particularly relevant in alopecia, where sustained biological activity is often required to regulate chronic inflammatory processes, maintain immunomodulatory effects, and support hair follicle regeneration (4). Inadequate drug exposure may limit the ability to continuously suppress pro-inflammatory signaling, preserve hair follicle immune privilege, or sustain activation of regenerative pathways involved in stem-cell activity, angiogenesis, and follicular remodeling (9, 13, 14). Consequently, delivery systems capable of providing controlled, prolonged release are increasingly viewed as essential to achieving durable therapeutic outcomes in alopecia treatment.

**Figure 1:**
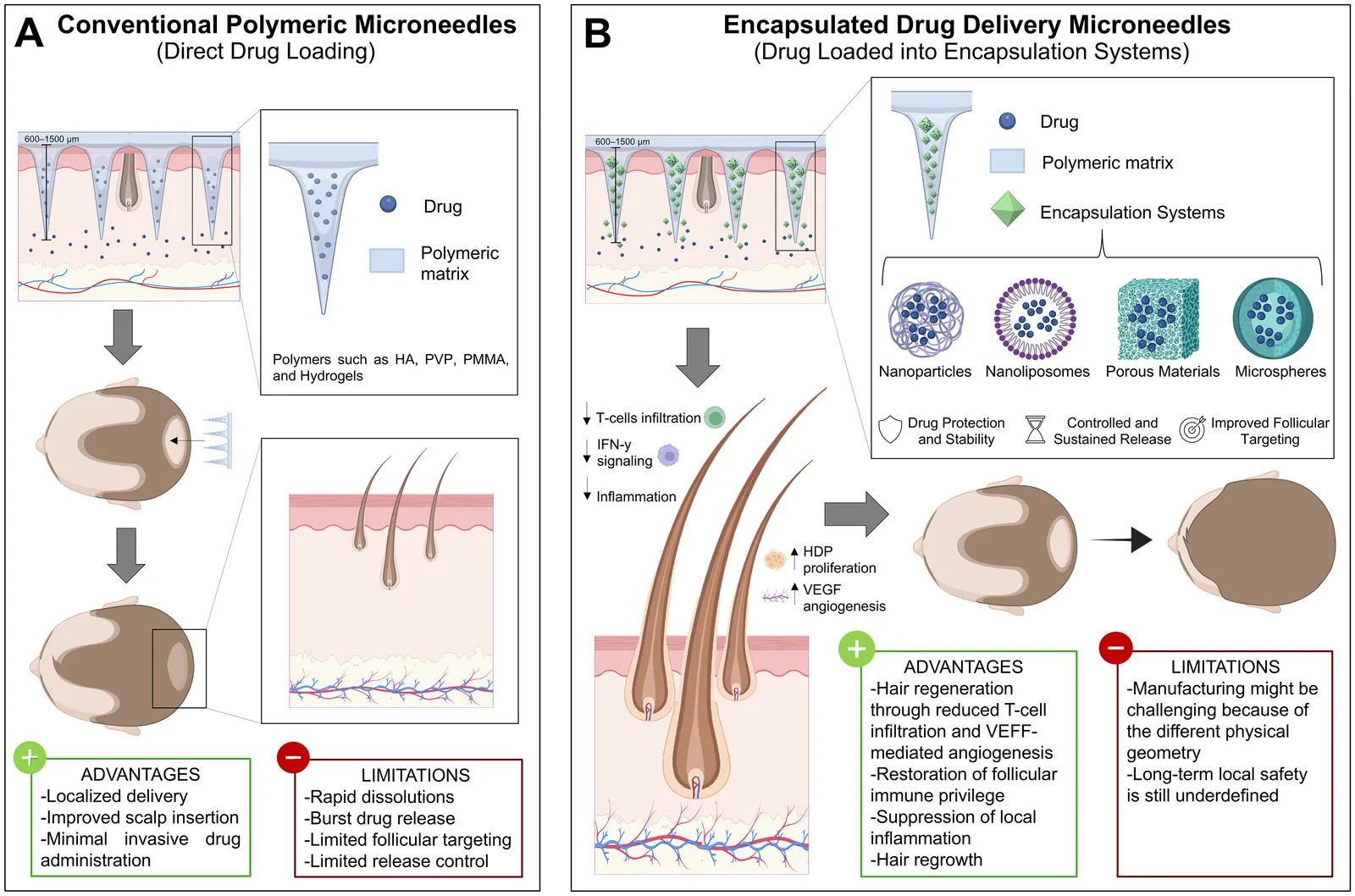
Conceptual comparison between **(A)** conventional polymeric microneedles and **(B)** encapsulation-integrated microneedle platforms for alopecia treatment. Created in BioRender.

To address these limitations, emerging microneedle platforms have increasingly incorporated advanced encapsulation systems (**Figure 1B**; **Table 1**), including nanoparticles, nanoliposomes, porous materials, and microspheres, as well as emerging technologies like light-guiding systems, to achieve more controlled and sustained drug release profiles and overcome rapid burst release associated with conventional designs (17). In these approaches, therapeutic agents are first loaded into carrier systems before being incorporated into the microneedle matrix. This hierarchical encapsulation strategy improves drug stability, protects bioactive molecules from premature degradation, and enables more precise modulation of release kinetics, thereby prolonging therapeutic activity within the follicular microenvironment (9, 18, 19). Beyond improving drug release control, these systems are increasingly being designed to actively regulate biological processes involved in hair regeneration and immune modulation, while maintaining localized therapeutic concentration over extended periods and enhancing treatment efficacy (9, 19).

**Table 1:** Comparative overview of conventional, encapsulation-integrated, and emerging microneedle platforms for alopecia treatment.

System	Delivery platform	Encapsulation strategy	Therapeutic agent	Alopecia type	Mechanism of action	Drug loading capacity	Release Profile	Advantages over conventional MNs	References
Conventional Polymeric Microneedle	Dissolving HA Microneedles	Traditional MN (Direct drug loading into HA matrix)	MXD	Chemo-induced alopecia	Follicular regeneration through CD44/Akt-mediated HDP proliferation, migration, and aggregation	6 mg MXD per patch (~0.1 mg in MN tips)	Applied for 2h, ~95% cumulative permeation within 24h	Traditional MN Benefits: enhanced follicular regeneration with minimal drug dose	(24)
	Shooting-Type Candlelit Dissolving Microneedles (HA/PVP)	Traditional MN (Direct drug loading into HA/PVP matrix)	TA	AA	Local immunomodulation through suppression of IFN-γ-mediated inflammatory signaling and T-cell activation	Not reported (TA formulation: 22.6% w/v)	Microneedle dissolution-mediated release (time not reported)	Traditional MN Benefits: improved scalp insertion and localized anti-inflammatory delivery	(23)
	PMMA MN Patch	Traditional MN (Drug infusion through PMMA microneedle-generated microchannels)	TA	AA	Enhanced intradermal corticosteroid delivery for local inflammation control and hair regrowth	Not reported (TA formulation: 5 mg/mL)	Complete corticosteroid absorption (time not reported)	Traditional MN Benefits: reduced pain compared with intralesional injections	(25)
	Hydrogel MN	Traditional MN (Direct loading of immunomodulators into hydrogel matrix)	IL-2 + CCL22	AA	Restoration of hair follicle immune privilege through local Treg recruitment and expansion	100 ng CCL22 + 10 ng IL-2 per patch	Sustained local delivery (release profile not explicitly reported)	Traditional MN Benefits: sustained local immunomodulation	(22)
Nanoparticle-Integrated Microneedle	PLGA nanoparticle-integrated dissolving microneedles (HA/BSP)	Polymeric nanoparticles	TFB	AA	Hair follicle regeneration through sustained JAK–STAT inhibition, reduced T-cell infiltration, and BSP-mediated angiogenesis	114.33 ± 0.03 μg of TFB per 10 x 10 MNP array	Sustained drug release for up to 48 h	Improved drug retention, sustained therapeutic activity, reduced dosing frequency, and enhanced immunomodulation compared with conventional microneedles	(43)
	PHA nanoparticle-integrated hydrogel forming microneedles (HAMA/PVA)	Polymeric nanoparticles	VEGF incorporated into the hydrogel matrix, and ritlecitinib encapsulated within PHA nanoparticles	AGA	Hair follicle regeneration through sustained JAK3/TEC inhibition, suppression of CD8^+^ T-cell and NK-cell activity, and VEGF-mediated angiogenesis.	10 mg of ritlecitinib per 100 mg of PHA nanoparticles	Long-term release over 28 days, characterized by an initial burst release during the first 7 days, followed by sustained release for the remaining 21 days	Provides sustained ritlecitinib release, improved mechanical strength, accelerated follicular activation, and enhanced angiogenic and regenerative responses compared with conventional hydrogel microneedles	(39)
Nanoparticle-Integrated Microneedle	Nanostructured lipid carrier–integrated dissolving microneedles (HA) combined with cold atmospheric plasma	Lipid-based nanoparticles	MXD	AGA	Hair follicle regeneration through enhanced minoxidil delivery, VEGF-mediated neovascularization, and NO-induced perifollicular vasodilation	62.68 μg of MXD per 10 × 10 MNP array	Continuous drug release for up to 24 h	Enhanced follicular drug delivery, angiogenesis, and perifollicular vasodilation, resulting in improved follicular proliferation and hair regeneration compared with blank microneedles	(50)
	Ferrum-chelated nanoparticles-integrated gas-propelled microneedles	Metal-chelated nanoparticles	Quercetin and puerarin	AGA	Hair follicle regeneration through Ferrum-chelated quercetin/puerarin-mediated ROS scavenging, suppression of NF-κB/MAPK inflammatory signaling, downregulation of senescence-associated genes (p16, p21, p53, and TGF-β1), reduced SRD5A2 expression, and activation of VEGF/β-catenin-associated angiogenesis and anagen-phase transition	41.02 ± 6.12 μg of nanoparticles per 12 × 12 MNP array	Rapid nanoparticle release, achieving complete release within 15 min	Increased drug delivery to deep follicular compartments through gas-propelled transport, resulting in improved ROS minimization, angiogenesis, and hair follicle rejuvenation compared with passive microneedles	(55)
Nanoliposome-Integrated Microneedle	Dissolving HA microneedles integrated with codelivery nanoliposomes (KK-NLPs@MNs)	Nanoliposomes (kopexil + kopyrrol co-encapsulated in a single lipid vesicle), then embedded in HA needle matrix	Kopexil + kopyrrol (MXD-like analogs)	AGA	Vesicles internalized by dermal papilla cells; co-delivered actives synergistically upregulate Ki67, β-catenin (Wnt), VEGF, and CD31, driving proliferation, peripheral angiogenesis, antioxidant effects, and telogen→anagen transition	In nanoliposomes: kopexil drug loading 6.64% (EE 97.6%), kopyrrol 3.31% (EE 97.4%); loaded at ~48.8 + 24.4 mg/mL (kopexil:kopyrrol 2:1). After embedding in HA needles, EE falls to ~61.8%/63.3%	Decelerated, sustained vs. free drug. KK-NLPs@MNs release ~55.8% (kopexil) and 54.5% (kopyrrol) cumulatively at 12 h, vs. a free-drug burst of 72.8%/79.7% within the first 4 h. Needles dissolve in skin within ~5 min	Co-encapsulates two actives at fixed ratio; stabilizes labile actives during fabrication; intradermal depot delivery achieving regrowth equal/superior to MXD at much lower dose with lower toxicity vs. free drug	(62)
	Dissolving composite HA/Bletilla striata polysaccharide (HA/BSP) microneedles loaded with cholesterol-free liposomes (Rg3-MNs)	Cholesterol-free ginsenoside Rg3 liposomes (Rg3 substitutes for cholesterol as bilayer-forming component), embedded in HA/BSP matrix	Ginsenoside Rg3	TE and AGA	Cholesterol-free design avoids cholesterol→testosterone→DHT pathway; Rg3 stabilizes vesicle and is bioactive; treatment raises VEGF, CD34, Ki67 and upregulates Wnt3a/Wnt10b, promoting angiogenesis, progenitor proliferation, and anagen induction	Rg3 encapsulation efficiency 93.66% in cholesterol-free liposomes; Rg3 content 127.13 ± 0.67 µg per MN patch (EE 89.55% in the patch)	~900 µm needles dissolve within ~30 min. Sustained transdermal release: 56.68% at 10 h rising to 83.87% at 72 h for Rg3-MNs, vs. only 14.27% at 72 h for free Rg3 liposomes (~6×). Dosing every 3 days (vs. daily MXD)	~900 μm needles dissolve within 30 min; cumulative transdermal release 83.87% at 72 h vs. 14.27% for free liposomes (~6×); dosing every 3 days matches daily MXD while improving hair density/quality	(65)
Porous-Integrated Microneedle	Composite PLA-substrate/HA microneedle patch with porous UiO-66 reservoir (MXD@UiO-66-MNs)	MOF depot: MXD adsorbed into porous UiO-66 (Zr-based MOF) particles loaded into HA needle tips on a PLA backing	MXD	AA	HA needles dissolve rapidly to deposit MXD-loaded UiO-66; MXD then releases slowly from the porous framework; acts via NF-κB pathway (↑ IκBα, ↓ phospho-P65), suppressing local inflammation; UiO-66 is intrinsically antibacterial	MXD loaded into UiO-66 at 21.9 wt% (mass fraction of MXD in MXD@UiO-66). MN patches formulated at 0.1–2 wt% MXD content (~650–13,000 ppm initial MXD)	Continuous, slow MXD release over 28 days (PBS, pH 7) via gradual UiO-66 degradation; higher loads release a larger cumulative fraction (e.g., 87.2% of initial load by day 28 for the 2 wt% patch; ~68–84% of maximum released in the first 14 days)	Decouples rapid insertion from slow release; sustained MXD release up to 28 days; independently tunable needle vs. depot phases; reduces dosing frequency and infection/aesthetic burden of wear	(71)
	Dual-continuous microneedle patch: HA drug needles + hydrogel sensor needles (theranostic)	pH-responsive MOF: glycyrrhizic acid adsorbed into ZIF-8 (Zn-imidazolate MOF), i.e. GA@ZIF-8 (“licorzinc”); acid-labile framework releases payload in inflamed tissue	Glycyrrhizic acid (licorzinc)	AA	GA@ZIF-8 reduces CD8+NKG2D+ T-cell infiltration, inhibits JAK activation, and lowers IL-15 and IFN-γ, interrupting the IFN-γ/IL-15 cytotoxic loop and helping restore follicular immune privilege; Eu3+–terpyridine hydrogel needles sense Zn2+	GA loading capacity of GA@ZIF-8 = 50.89 ± 0.24 wt% (optimal GA: ZIF-8 5:1, mixed 2 h at 35 °C)	pH-responsive. 24 h cumulative GA release from GA@ZIF-8 = 71.7% at pH 7.4 vs. 98.8% at pH 5.4 (free GA only ~51–53%); acidic release rate ~30× faster. Drug needles dissolve in ~2 min	Combines therapy + diagnostics in one device; pH-triggered release localized to inflamed tissue; drug needles dissolve in 2 min; real-time Zn2+ sensing (10^-^^8^–10^-^¹ M) with minimal ionic interference	(70)
	Magneto-responsive composite microneedles (mesoporous iron oxide nanoraspberry + PVA), 3D-printed mold	Mesoporous iron oxide nanoraspberry (MIO) captures hydrophobic drug; embedded in dissolvable PVA needles	MXD	AGA	High-surface-area mesoporous iron oxide adsorbs MXD; external magnetic field induces mild local heating that triggers on-demand release and increases cutaneous blood flow/vasodilation, promoting hair regrowth	MXD loading efficiency in the mesoporous iron-oxide nanoraspberry (MIO) ~ 88% (loaded from a 1% Mx feed).	Magnetically (heat)-triggered, on-demand. Passive release ~20% of Mx by 120 min; with a 1-min magnetic field, ~23% releases within 10 min and >40% over 120 min (PBS, 37 °C)	Adds remote (magnetic/thermal) on-demand triggering absent in passive microneedles; controlled, externally gated release; reduces off-target exposure	(69)
	Degradable nanocomposite microneedle patch (ZCQ/MN) with Zn/Cu dual-doped mesoporous silica	Mesoporous silica nanospheres dual-doped with Zn/Cu and loaded ZCQ; released as needles dissolve	Quercetin + Cu2+ + Zn2+	AGA	Multi-target: quercetin suppresses inflammation/oxidative stress and DHT attack; Cu2+ drives angiogenesis; Zn2+ supports follicle stem-cell activity and inhibits 5α-reductase/DHT signaling (simultaneously addressing the main AGA pathways)	Quercetin loading in Zn/Cu dual-doped mesoporous silica (ZC-MSN) ~241 mg/g, vs. ~81 mg/g for undoped MSN (~3× higher), enabling multi-agent codelivery.	MN swells/breaks within 12 h and fully dissolves by 24 h. Sustained release of quercetin, Zn²^+^ and Cu²^+^ over 14 days (cumulative at 14 d: Zn²^+^ 4.87, Cu²^+^ 5.22, SiO_3_²^-^ 80.53 µg/mL; Qu 2.81–6.53 µg/mL).	Very high, tunable loading of mesoporous silica enables true multi-agent codelivery; systems-level (not single-target) intervention from one patch	(74)
	3D-printed dissolving microneedles with detachable, pH-responsive tips (recyclable deep-eutectic-solvent based)	ZIF-8-encapsulated MXD in detachable, acid-responsive needle tips	MXD	AGA	Acid-labile ZIF-8 enables inflammation-localized, pH-triggered MXD release; detachable tips act as retained intradermal depots	MXD in ZIF-8: encapsulation efficiency 89.6%, drug-loading efficiency 9.1%; ~122 µg MXD per patch (≈3.39 µg per needle)	pH-triggered (acid-labile ZIF-8). MXD release ~80% within 24 h at pH 5.0 and ~58% at pH 6.5, but negligible at pH 7.0. Zn²^+^ cumulative ~78%/45%/5% at pH 5.0/6.5/7.0 by ~53 h. First-order kinetics at pH 5.0.	Detachable depot tips + 3D-printed/recyclable manufacturing for practical, scalable application; stimulus-responsive release	(75)
Microsphere-Integrated Microneedle	Dissolving MN patch with DUT-loaded PLGA MSs	DUT encapsulated in PLGA MSs and embedded in a HA/PVP tip matrix.	DUT	AGA	Local 5α-reductase inhibition; reduced testosterone-to-DHT conversion, intradermal DHT levels, and TGF-β1 expression	14.3 ± 0.7% DUT loading in the MSs and 51.2 ± 5.54 μg DUT per patch.	Limited initial release (~9.6% within 12 h), followed by sustained release reaching ~90.8% over approximately 20 days.	Sustained local DUT release, reduced dosing frequency, and maintained therapeutic activity between applications	(21)
	Dissolving PVA/sucrose MN patch with MXD-loaded PLGA MSs	MXD encapsulated in PLGA MSs and loaded into dissolving PVA/sucrose microneedles	MXD	AGA	Sustained follicular stimulation; increased hair coverage, hair diameter, hair density, follicle length, dermis thickness, and Ki-67 staining	56.3 ± 9.2 μg MXD per patch	Almost no initial burst release; approximately 90% released within 14 days, with complete release occurring at around 1 month	Sustained intradermal MXD release, improved local delivery efficiency, reduced administration frequency, and stronger regrowth response with lower drug amount	(87)
Light-Guiding Microneedle	Light-Guiding Microneedle Patch(PMMA)	Light-guiding MNs (no drug loading)	Red (633 nm) and Green (522 nm) LED	AGA	Enhanced cellular metabolism, fibroblast proliferation, wound-healing responses, VEGF upregulation, reduced inflammation, improved local blood circulation, and direct stimulation of deep follicular structures through increased light penetration			Red light improves hair density, and its wavelength penetrates the skin more deeply than other visible wavelengths. Green light supports tissue repair and cell migration.	(102)

Collectively, these systems illustrate how advanced microneedle-based encapsulation strategies are evolving from passive drug delivery platforms toward multifunctional systems capable of simultaneously enhancing transdermal transport, promoting follicular regeneration, and modulating local immune responses relevant to alopecia pathophysiology. While several reviews have discussed microneedle-mediated drug delivery for alopecia, most have primarily focused on microneedle fabrication, transdermal delivery performance, and therapeutic outcomes (4, 5, 13). Despite the growing body of literature on microneedle-mediated drug delivery for alopecia, a comprehensive framework describing how encapsulation-integrated microneedle platforms coordinate controlled drug delivery, immunomodulation, and hair follicle regeneration through engineering of the follicular microenvironment is still lacking. This review addresses that gap by systematically examining current encapsulation strategies and their biological implications.

## 2 Conventional polymeric microneedle platforms for alopecia treatment

Polymeric systems are widely integrated into microneedle-based drug delivery platforms, where they serve as both structural frameworks and drug carriers (20). Hyaluronic acid (HA), polyvinylpyrrolidone (PVP), polymethyl methacrylate (PMMA), and other hydrogel-forming polymers are used because of their biocompatibility, mechanical properties, and ability to regulate delivery (21–24). Each material offers a distinct advantage: HA can contribute to dermal papilla cell migration and follicular regeneration (24). PVP can improve mechanical strength and control dissolution rates (23), and PMMA provides the rigidity needed to create microchannels for subsequent drug diffusion (25).

Polymer selection plays a critical role in determining microneedle performance, as material composition directly influences mechanical strength, drug-loading capacity, and release behavior, which in turn affects therapeutic outcomes in alopecia treatment. These include not only drug delivery efficiency but also downstream biological responses such as follicular targeting, local immunomodulation, and hair regeneration. Despite these advantages, polymer-based microneedle systems remain limited by challenges in achieving fully controlled and sustained drug release under complex biological conditions. These limitations are further explored following the overview of individual polymer platforms, as they motivate the development of advanced encapsulation strategies to improve drug stability, release kinetics, and therapeutic precision within the follicular microenvironment.

### 2.1 Dissolving polymeric microneedle systems

Among the various polymers investigated for microneedle fabrication, HA has emerged as one of the most extensively studied for alopecia treatment due to its excellent biocompatibility, biodegradability, and intrinsic role within the extracellular matrix (ECM) of the skin and hair follicle microenvironment (24, 26, 27). HA is naturally abundant in the dermis, where it contributes to tissue hydration, matrix organization, cell migration, and intercellular signaling, all of which are important for maintaining normal hair follicle function and regeneration. Its high hydrophilicity promotes water uptake and rapid dissolution in the skin environment, facilitating the release of incorporated therapeutics (26, 27). Notably, reduced HA levels have been associated with hair loss progression and follicular dysfunction, suggesting that restoration of HA-rich microenvironments may support regenerative processes within the scalp (24, 26–28).

Beyond its role as a structural biomaterial, Kim et al. further investigated whether the intrinsic regenerative properties of HA could be combined with localized drug delivery as a dissolving microneedle. The authors fabricated dissolving microneedle arrays composed entirely of HA, consisting of 76 needles approximately 600 μm in height and loaded with MXD. Following insertion into the skin, the hydrophilic HA matrix gradually dissolved over approximately 2 h, enabling direct drug deposition within the dermal and perifollicular regions while bypassing the stratum corneum barrier. The microneedles exhibited a drug-loading capacity of approximately 6.7% (w/w) and achieved nearly 95% cumulative MXD permeation within 24 h, demonstrating substantially improved transdermal delivery compared with conventional topical administration (25).

Mechanistically, these regenerative effects were associated with activation of signaling pathways involved in cell proliferation and follicular development. HA treatment increased CD44 expression by approximately 51% and induced more than a twofold increase in Akt phosphorylation, resulting in enhanced expression of proliferation-related markers such as cyclin D1 (24, 29). CD44 is a principal receptor for HA and plays an important role in mediating cell–matrix interactions, while Akt signaling regulates cell survival, proliferation, and tissue regeneration. Together, activation of the CD44/Akt axis provides a mechanistic explanation for the enhanced proliferative and regenerative responses observed in human dermal papilla cells following HA exposure (24, 29). These findings suggest that HA contributes to hair follicle regeneration not only as a drug-delivery matrix but also as a biologically active material capable of promoting pro-regenerative signaling within the follicular microenvironment.

The therapeutic efficacy of the system was evaluated in a chemotherapy-induced alopecia mouse model and compared with conventional topical MXD treatment. The MXD-loaded HA microneedles induced significantly greater hair regrowth despite delivering only approximately 10% of the dose administered through the topical formulation (24). Histological analysis revealed enhanced recovery of hair follicles, increased cellular proliferation within the follicular compartment, and a greater proportion of follicles entering the anagen growth phase. These findings suggest that the enhanced therapeutic response may reflect the combined effects of improved local MXD delivery and the intrinsic regenerative properties of HA observed *in vitro*. Collectively, this study demonstrates that HA can function simultaneously as a structural material, a drug-delivery matrix, and a biologically active component that promotes hair follicle regeneration, highlighting its potential as a multifunctional biomaterial for microneedle-based alopecia therapies (24).

Composite polymer systems have likewise been explored to enhance mechanical stability, optimize dissolution behavior, and improve localized drug delivery within the scalp microenvironment (23, 27). By combining polymers with complementary physicochemical properties, these platforms can be tailored to address specific therapeutic challenges associated with different forms of alopecia (28). This is particularly relevant in AA, where hair follicle damage is driven by autoimmune inflammation, collapse of immune privilege, and infiltration of cytotoxic T cells around the follicular bulb (6, 30). In this context, Lee et al. developed a shooting-type candlelit dissolving microneedle system composed of HA and PVP for localized delivery of TA, a corticosteroid used to suppress inflammatory responses in AA (23).

The use of HA and PVP was strategically selected to balance biocompatibility, dissolving behavior, and mechanical performance. HA served as a biocompatible dissolving matrix, while PVP was incorporated to improve formulation viscosity, structural integrity, and drug-loading capacity during microneedle fabrication (23, 31). The final formulation contained TA (22.6% w/v), HA (45% w/v), and PVP (5% w/v), forming a viscous polymer-drug mixture suitable for fabrication of the candlelit microneedle geometry. Quantitative analysis showed that each 61-needle array loaded 1.20 ± 0.02 mg of TA. The candlelit design was selected because its expanded middle region allows a larger fraction of TA to be positioned within the microneedle body and delivered deeper into the dermis, where the inflamed hair follicles are located. The microneedles had a height of 918 ± 19 μm, a tip diameter of 37 ± 6 μm, and a head diameter of 300 ± 12 μm, dimensions intended to support dermal insertion and follicle-proximal drug deposition. In addition, the platform incorporated a patchless shooting-type applicator containing micropillars that individually pushed the microneedles into the scalp. This design directly addressed a practical challenge in alopecia treatment: hair can interfere with the adhesion and insertion of conventional microneedle patches, leading to inconsistent delivery (23).

Functionally, the system was designed to suppress the inflammatory microenvironment that drives AA. In this disease, IFN-γ-mediated signaling promotes the expression of chemokines such as CXCL9, CXCL10, and CXCL11, which recruit autoreactive T cells to the hair follicle and contribute to immune-mediated follicular damage (6, 30). To recapitulate this inflammatory environment *in vitro*, human outer root sheath (ORS) cells were cultured and stimulated with IFN-γ and poly(I), a synthetic double-stranded RNA analog that mimics viral danger signals and activates innate immune and inflammasome-related pathways associated with AA pathogenesis. Following induction of inflammation, cells were treated with dissolved TA-loaded candlelit dissolving microneedles at concentrations of 0.1 and 1 mg/mL and compared with untreated inflammatory controls (23).

Treatment with TCDs improved cell viability in the IFN-γ/poly(I)-stimulated model. Subsequent RT-qPCR also showed reduced expression of NLRP3, caspase-1, IL-1β, CXCL1, CXCL9, CXCL10, and CXCL11 (23), markers associated with inflammasome activation, inflammatory cytokine production, and immune cell recruitment in AA (23, 30). Changes in secondary lymphoid tissues were also observed: spleen and skin-draining lymph-node size decreased, and the splenic CD3^+^ T cell fraction fell from 37.46 ± 6.15% in untreated controls to 18.35 ± 3.25% after TCD treatment (23). Taken together, the coordinated reduction in these markers is consistent with attenuation of inflammatory pathways implicated in immune-mediated follicular damage, although the study did not establish the clinical significance of the systemic immune changes.

The therapeutic efficacy of the shooting-type candlelit dissolving microneedle (TCD) platform was subsequently evaluated in an AA-induced C3H/HeJ mouse model and compared with two clinically relevant treatment controls: topical TA cream and intradermal TA injection. Following three weeks of treatment, mice receiving the TCD platform exhibited visible skin pigmentation and more uniform hair regrowth than either control. Quantitative analysis further demonstrated significantly higher hair-cycle scores and increased interfollicular skin thickness in the TCD group, findings consistent with enhanced induction of the anagen growth phase and improved follicular recovery (23).

Together, these studies illustrate the regenerative and immunomodulatory potential of HA-based microneedles. However, because hydrophilic HA generally dissolves rapidly, simple HA matrices may be ill-suited to prolonged release; this limitation motivates more complex carrier architectures (28).

### 2.2 Non-dissolving polymeric microneedle systems

PMMA is a synthetic polymer widely employed in biomedical devices due to its mechanical strength, chemical stability, and biocompatibility (4, 9, 25). In contrast to biodegradable polymers frequently utilized in dissolving microneedle systems, PMMA is non-resorbable and remains structurally intact following cutaneous insertion. This structural integrity allows for effective penetration of the stratum corneum and avoids issues with fracturing that have been documented in softer microneedles (4). Accordingly, PMMA microneedle arrays have been investigated as a tool to enhance intradermal drug delivery for the management of both AA and AGA (25).

A clinical investigation of this approach in AA utilized PMMA microneedles of approximately 800 μm in length to transdermally deliver TA. This approach achieved approximately 76–77% reductions in SALT (Severity of Alopecia Tool) scores after three months, demonstrating efficacy comparable to conventional intralesional injections while significantly reducing treatment-associated pain (VAS score: ~4.0 vs. ~5.3) (32).

The mechanical microinjury induced by microneedle insertion may itself contribute to therapeutic benefit beyond drug delivery alone. Microneedle-induced microtrauma activates the Wnt/β-catenin signaling pathway, a key regulator of hair follicle stem-cell activation and hair follicle cycling, promotes neoangiogenesis around the hair follicle, and upregulates vascular endothelial growth factor (VEGF) and epidermal growth factor (EGF), both of which have been shown to stimulate the transition of follicles from telogen into the anagen phase and promote hair regrowth (4).

Despite these therapeutic benefits, the rigid polymer approach presents notable limitations that constrain its clinical utility. Given the non-biodegradable nature of PMMA, the microneedles function exclusively as penetration tools, creating microchannels in the stratum corneum without contributing to drug encapsulation or sustained release (25). Drug delivery therefore depends entirely on passive diffusion through the channels formed, affording limited control over intradermal dosing and introducing an additional procedural step involving the application of a topical formulation or patch following microneedle pretreatment. These constraints highlight the impetus for transitioning toward materials that integrate both robust penetration and drug-eluting capabilities.

### 2.3 Hydrogel-based polymeric microneedle systems

Hydrogels are crosslinked, three-dimensional polymeric networks with the capacity to absorb substantial quantities of aqueous fluid while maintaining structural integrity (4, 22, 33). They have several inherent benefits, including the ability to tune drug release rate via crosslink density and the ability to be removed intact following treatment (4). Significant interest has been placed in utilizing hydrogels for transdermal drug delivery due to their high water content and versatility in accommodating diverse therapeutic molecules (22, 34).

In hydrogel microneedle systems, therapeutic loading is integrated directly into the fabrication process: the active agent is homogeneously dispersed throughout the polymer solution prior to crosslinking, becoming physically entrapped within the resulting three-dimensional network. Following insertion into the cutaneous tissue, the hydrogel absorbs fluid and expands, allowing entrapped cargo to escape into the surrounding microenvironment. This mechanism was employed by Younis et al. (2024), who engineered a hydrogel microneedle patch composed of hyaluronic acid crosslinked with 8-arm polyethylene glycol (HA-8arm-PEG). The patch consisted of an array of 121 needle projections, each 600 μm in length, to deliver two immunomodulatory agents: CCL22 (a CC-motif chemokine ligand that functions as a potent chemoattractant for regulatory T cells) and IL-2, an interleukin cytokine that serves as a key Treg survival and proliferation factor, loaded at 100 ng patch^-^¹ and 10 ng patch^-^¹, respectively. In a murine model of immune-mediated alopecia, this combination significantly expanded Treg populations within lesional skin compared with empty-patch and untreated controls (5.9% vs. 1.36% and 0.47% of CD45^+^ cells, respectively), indicating successful local recruitment and expansion of immunosuppressive Tregs capable of restoring immune tolerance within the follicular microenvironment. This increase was accompanied by reduced infiltration of cytotoxic CD8^+^ T cells, suppression of hallmark AA inflammatory signaling, and sustained hair regrowth beginning at week 3 of treatment, demonstrating that the platform effectively re-established the immune privilege required to support hair follicle regeneration (22).

In terms of inflammatory signaling, the IFN-γ response pathway was 2.33-fold less enriched, and the IL-6–JAK–STAT3 signaling pathway was correspondingly suppressed, in lesions treated with IL-2+CCL22-loaded microneedles relative to untreated controls, as determined by RNA sequencing and gene set enrichment analysis. Because IFN-γ signaling is essential for AA development and persistence, this downregulation reflects a direct attenuation of the autoimmune cytotoxic program driving the disease, rather than a generic anti-inflammatory effect (22). Notably, when the same IL-2 and CCL22 doses were delivered by conventional subcutaneous injection rather than through the microneedle patch, no comparable improvement in hair regrowth was observed. The authors attribute this difference to two complementary mechanisms specific to the microneedle platform. First, the microtrauma generated by the needles themselves stimulates local release of growth factors such as VEGF and platelet-derived growth factor, which enhance perifollicular blood flow independently of the immunomodulatory cargo. Second, the microneedle architecture distributes the drug more homogeneously throughout the dermal milieu surrounding the follicle, whereas a bolus subcutaneous injection concentrates the drug in a single depot and fails to achieve the same distribution across the affected lesion (22).

Nevertheless, hydrogel systems have important constraints. Their lower elastic modulus relative to rigid thermoplastics can complicate consistent penetration of thicker or fibrotic skin, and some configurations require structural reinforcement (22). Release also depends on crosslinking density, polymer concentration, and local tissue hydration, which can vary among patients (22). These mechanical and release-variability constraints motivate carrier-based designs in which payload protection and release are governed partly by the encapsulating phase rather than by the bulk hydrogel alone.

## 3 Nanoparticle-integrated microneedle systems for alopecia treatment

Nanoparticles represent a promising class of delivery systems that can address key limitations of conventional polymer-based microneedle platforms, particularly in drug protection and release control (35). Their functionality results from their surface properties, and structural architecture, which together determine their stability, biodistribution, and drug-release behavior (18). Broadly, nanoparticles (NPs) can be classified into three main categories: polymeric NPs, lipid-based NPs, and inorganic NPs, each offering different advantages in terms of biocompatibility, drug loading capacity, and release profiles. In addition, NPs can support targeted delivery through surface modification, helping improve site-specific accumulation (36). These attributes make NPs attractive for alopecia treatment, where effective therapy often requires the localized and sustained delivery of bioactive agents to the hair follicle microenvironment (37).

As illustrated in **Figure 2**, NP-integrated microneedle systems can deliver polymeric, lipid-based, and metal-chelated (inorganic) NPs directly to the follicular microenvironment, where they protect the therapeutic cargo and enable localized, controlled drug release (38). Depending on the therapeutic strategy, NPs can facilitate the delivery of anti-inflammatory compounds, immunomodulatory agents, growth factors, nucleic acids, and other regenerative molecules with the potential to reduce systemic exposure. When combined with microneedles, these systems improve follicular targeting and prolong drug residence within the scalp, supporting both immune regulation and hair follicle regeneration (39). To better illustrate the design strategies and therapeutic advantages of NP-microneedle integrated systems, the following sections examine the major NP classes explored for alopecia treatment.

**Figure 2:**
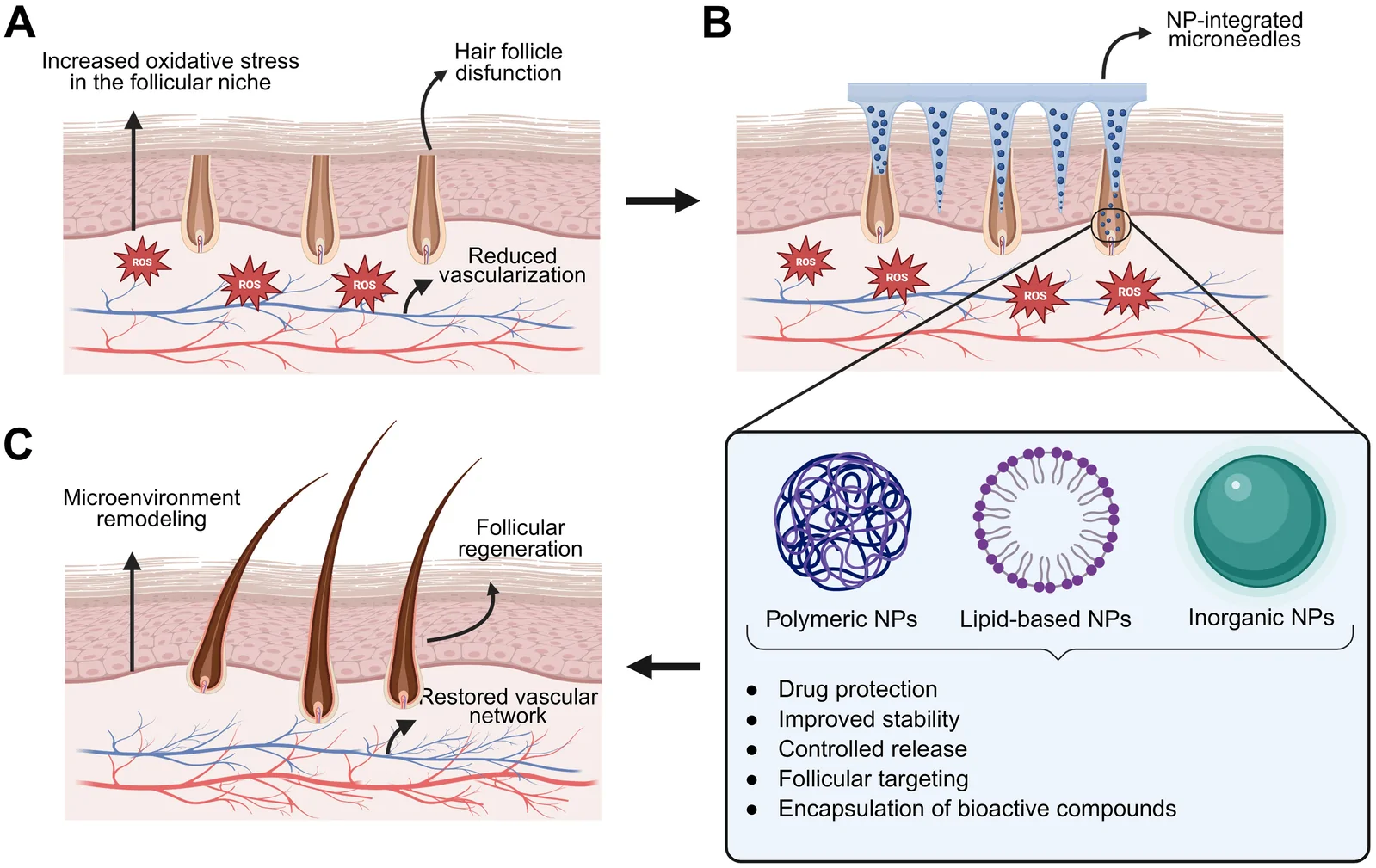
Schematic representation of nanoparticle-integrated microneedle systems for alopecia treatment. **(A)** Follicular Microenvironment Alterations in Alopecia. **(B)** Nanoparticle (NP)-Integrated Microneedle-Mediated Delivery. **(C)** Follicular microenvironment restoration. Created in BioRender.

### 3.1 Polymeric nanoparticle–microneedle systems for alopecia treatment

Polymer-based NPs are popular drug delivery platforms that, through their biocompatibility and tunable properties, can enhance drug stability, enable controlled release and targeted delivery, and reduce systemic toxicity (40). These NPs usually range from 10 to 1000 nm and can be fabricated through a variety of approaches such as polymerization, self-assembly of copolymers into micelles, emulsification-based formulations and electrospinning (41, 42). Depending on their source, the polymers used for fabrication may be synthetic or natural, each offering distinct properties to the resulting NPs. Synthetic polymers offer precise control over physicochemical properties and degradation behavior, while natural polymers enable biocompatibility, low immunogenicity and bioactivity (41). In the context of alopecia, polymeric NPs can facilitate sustained follicular delivery of bioactive compounds, supporting prolonged therapeutic activity and reducing the need for frequent administration.

Poly(lactic-co-glycolic acid) (PLGA) is one of the most widely investigated polymers for NP-based drug delivery due to its biodegradability, biocompatibility, and well-established safety (6). In addition, its tunable degradation kinetics enable drug release profiles to be tailored over time, while its ability to encapsulate both hydrophilic and hydrophobic compounds makes it a versatile carrier for a wide range of therapeutic agents (6). A recent study conducted by Zhao and colleagues highlighted these properties by incorporating TFB-loaded PLGA NPs into dissolvable microneedles for alopecia treatment (43). In this system, TFB, a JAK inhibitor used to promote hair regeneration, is first encapsulated within PLGA NPs and subsequently integrated into a microneedle matrix composed of HA and *Bletilla striata* polysaccharide (BSP) at a 2:3 (HA: BSP, w/w) ratio. The resulting NPs exhibited a drug loading of approximately 23% and an encapsulation efficiency of ~93% while each 1 × 1 cm microneedle patch delivered a total of ~114 μg of TFB. Compared with conventional TFB-loaded microneedles, in which the drug is directly embedded within the microneedle matrix and released rapidly upon dissolution, the PLGA NP-microneedle delivery system extends drug release for up to 48 hours. This sustained release profile offers the potential for a longer-acting therapy while reducing dosing frequency. The controlled release is governed by the PLGA core, which acts as a diffusion barrier. As a result, a small fraction of TFB is released rapidly during the initial burst, while the remaining drug is released progressively through diffusion and polymer degradation (19). This staged release behavior prevents early drug exhaustion and supports prolonged activity in the hair follicles after microneedle dissolution.

Beyond providing sustained drug release, the PLGA NP formulation enhanced the immunomodulatory activity of TFB by maintaining prolonged drug exposure within the follicular microenvironment, achieving more sustained inhibition of the inflammatory cascade than microneedles containing TFB directly dispersed within the microneedle matrix (non-encapsulated). This sustained local delivery is particularly important in AA, where pro-inflammatory cytokines such as IFN-γ and IL-15 contribute to the activation of autoreactive T cells that target hair follicles (44). Consistent with this mechanism, TFB NP-MNs reduced IFN-γ levels from approximately 220 pg/mL in untreated mice to below 80 pg/mL and decreased IL-15 concentrations from approximately 70 pg/mL to 25 pg/mL. Because these cytokines trigger the inflammatory immune response against hair follicles, their reduction helps suppress disease progression and creates a more favorable environment for hair regrowth (45). These reductions were accompanied by lower CD4^+^ T-cell levels in mouse skin and spleen tissues collected at the study endpoint, suggesting decreased immune-cell activation and reduced inflammatory signaling around hair follicles. Together, these findings indicate that the sustained-release PLGA NP system prolongs drug availability while creating a more favorable follicular microenvironment for hair regeneration (43).

These results further emphasize that the therapeutic performance of NP-microneedle systems depends not only on sustained drug release but also on the physicochemical properties of the polymeric carrier. Accordingly, additional biodegradable polymers have been explored to provide complementary advantages for alopecia treatment beyond sustained release, including enhanced mechanical performance and regenerative activity. Among these, polyhydroxyalkanoate (PHA) NPs have emerged as a platform for JAK inhibitor delivery through microneedle-based systems (39). PHAs are a family of biodegradable and biocompatible polyesters produced by microorganisms that have been investigated as NP carriers for drug delivery applications because of their ability to efficiently encapsulate hydrophobic compounds and provide sustained drug release (46). In the study by Ding and colleagues, PHA NPs encapsulating ritlecitinib, a JAK inhibitor, were prepared using a 1:10 (drug: polymer, w/w) ratio and subsequently integrated into hydrogel-forming microneedles fabricated from 1% methacrylated hyaluronic acid (HAMA). The platform also incorporated VEGF as a complementary regenerative cue, while a 5% polyvinyl alcohol (PVA) backing layer provided structural support to the patch (39).

This NP-integrated microneedle platform combined the complementary advantages of hydrogel-forming microneedles and PHA NPs: the microneedles provided minimally invasive delivery across the stratum corneum and localized deposition within the scalp, while the PHA NPs enabled the encapsulation and sustained release of the hydrophobic JAK inhibitor (39). Compared with blank hydrogel microneedles, the incorporation of ritlecitinib-loaded PHA NPs improved both the delivery performance and therapeutic activity of the platform. The PHA NPs enabled sustained release of the JAK inhibitor, with approximately 69% of the encapsulated drug released over 28 days. In addition, NP incorporation reduced tip fracture from approximately 12% to 3.5% under a 200 g load, improving microneedle penetration and enabling more reliable drug delivery to the hair follicle. Functionally, this translated into improved follicular activation, as melanin deposition appeared by day 7 in treated mice compared with day 10 in the blank microneedle group. The incorporation of VEGF as a complementary regenerative cue promoted endothelial cell migration and angiogenesis, while the sustained release of ritlecitinib contributed to improving the perifollicular immune microenvironment. Consistent with these mechanisms, HUVEC migration increased to 68.3% after 24 h and 96.2% after 48 h, supporting vascular remodeling around the hair follicles and creating conditions favorable for hair regeneration.

Although polymeric NPs have shown considerable functionality for microneedle-mediated alopecia therapy, they represent only one class of the NPs being explored. Lipid-based NPs have also been incorporated into microneedles to benefit from their physicochemical properties and expand the range of nanocarriers available for transdermal therapeutic delivery.

### 3.2 Lipid-based nanoparticle–microneedle systems for alopecia treatment

Lipid-based NPs provide an alternative strategy for the encapsulation and controlled delivery of therapeutic agents, particularly poorly water-soluble compounds. The two major classes of lipid-based nanocarriers are solid lipid NPs (SLNs) and nanostructured lipid carriers (NLCs) (47). SLNs are first-generation lipid-based NPs typically ranging from 50–100 nm that consist of a solid lipid matrix stabilized by surfactants, providing protection against chemical and enzymatic degradation. However, their limited drug-loading capacity and tendency to expel drugs during storage led to the development of second-generation NLCs, which combine solid and liquid lipids within the core. Unlike SLNs, this mixed lipid composition creates an imperfect crystal lattice that increases drug-loading capacity, improves physical stability, and maintains drug retention over time (48). This lipid organization also protects the encapsulated drug from premature degradation while maintaining close interaction with the skin, supporting controlled drug release and enhancing local drug delivery (49). Such properties are advantageous for alopecia treatment, in which sustained drug exposure at the target site is required to address the key pathogenic mechanisms involved in hair loss.

Chen and colleagues exemplified this approach by developing an integrated therapeutic platform combining MXD-loaded nanostructured lipid carriers (MXD-NLCs), dissolvable microneedles, and cold atmospheric plasma (CAP) (50). The system integrates the complementary functions of each component, with microneedles enabling localized drug delivery, NLCs improving drug stability and sustained MXD release, and CAP contributing to modulation of the follicular microenvironment. Consequently, this approach extends beyond simple drug delivery optimization by targeting multiple aspects of AGA pathogenesis simultaneously (50). The MXD-NLCs were prepared using glyceryl monostearate and squalene as the solid and liquid lipid phases, respectively, with Poloxamer 188 as the stabilizing surfactant. The NPs achieved an encapsulation efficiency of 80.22% and contained approximately 30.66 wt.% MXD. To concentrate drug delivery at the insertion site, the microneedle tips were fabricated from a mixture of 40 wt.% HA and 4 wt.% MXD-NLCs (10:1 HA: NLC, w/w), with each 1 × 1 cm² patch containing approximately 62.68 μg of MXD (50).

Transdermal delivery studies performed *in vivo* in a murine model, demonstrated that the MXD-NLC microneedle platform achieved approximately 22.02 μg/cm² of MXD delivery after 24 h, corresponding to a 2.7-fold increase relative to MXD tincture (a topical solution of MXD dissolved in propylene glycol, alcohol, and water without NP encapsulation or microneedle-assisted delivery). Additionally, drug retention within the skin reached 17.21 μg/cm², 11-fold higher than the conventional formulation (topical MXD tincture), highlighting the ability of the NP-microneedle system to localize and retain the therapeutic payload within the target tissue. Compared with topically applied MXD-NLCs, the microneedle platform also improved drug delivery by 1.82-fold, demonstrating the importance of microneedle-assisted transport in overcoming the skin barrier and maximizing local drug deposition (50).

The improved delivery achieved by the integrated MXD-NLC microneedle-CAP platform was accompanied by enhanced regenerative responses. Compared with untreated controls, the combined treatment produced a 17.5-fold increase in VEGF expression, exceeding the responses to MXD-NLC microneedles (15.2-fold), CAP alone, and conventional MXD tincture (4.3-fold). As a regulator of angiogenesis during the telogen-to-anagen transition, VEGF supports the vascular remodeling required for follicular growth (51). Histological and immunostaining analyses also indicated anagen entry and increased Ki-67 and CD31 staining (52), consistent with greater follicular proliferation and perifollicular angiogenesis (53).

Overall, these findings suggest that the integrated platform enhances hair regeneration through improvements in follicular morphology, angiogenesis, and cellular proliferation. However, because the study did not evaluate microneedles loaded with non-encapsulated MXD, the specific contribution of NLC encapsulation to the observed therapeutic effects remains difficult to determine. While polymeric and lipid-based NP-microneedle systems primarily improve drug delivery and sustained therapeutic activity, other NP platforms have been developed to directly modulate the oxidative microenvironment associated with hair loss.

### 3.3 ROS-responsive nanoparticle platforms for alopecia treatment

While the polymeric and lipid NP systems discussed in Sections 3.1 and 3.2 primarily focused on immunomodulation and restoration of the perifollicular microenvironment through JAK inhibition and angiogenic stimulation, another important contributor to AGA progression is oxidative stress-induced follicular aging (54). Oxidative stress, characterized by the accumulation of reactive oxygen species (ROS) in the perifollicular microenvironment, promotes dermal papilla cell senescence (aging of the papilla), shortens the anagen phase, and drives follicular miniaturization in AGA (54). Addressing these interconnected mechanisms requires delivery systems capable of reaching deep follicular compartments while simultaneously reducing oxidative stress and reversing senescence-associated molecular alterations.

To address these challenges, Chen and colleagues developed a gas-propelled microneedle platform that uses effervescent chemistry to propel iron-chelated puerarin and quercetin NPs (PQFN) into deep follicular compartments (55). The PQFN formulation consisted of iron-chelated quercetin combined with puerarin at a 2:1 mass ratio and stabilized with lecithin, while microneedles were fabricated using a 1:1 blend of PVP K30 and PVP K90. The system incorporated an effervescent backing layer containing citric acid and sodium bicarbonate in a 4:5 weight ratio, which generated carbon dioxide bubbles upon contact with interstitial fluid in the dermis, actively driving the NPs towards the hair follicle. Unlike conventional passive microneedles, which rely on diffusion and often leave most of the therapeutic payload within superficial skin layers, the gas-propelled platform increased delivery to the hair bulb region from approximately 35% to over 80% of follicles. In addition, PQFN exhibited potent antioxidant activity, achieving over 80% free radical scavenging at a NP concentration of 60 µg/mL.

These antioxidant properties translated into biological effects directly targeting the mechanisms underlying follicular aging. In testosterone-induced dermal papilla cells, PQFN effectively reduced intracellular and mitochondrial ROS, restored mitochondrial membrane potential and calcium homeostasis, and significantly decreased cellular senescence, indicating recovery of dermal papilla cell function rather than simple ROS neutralization. RNA sequencing further confirmed these effects by revealing suppression of inflammatory pathways, including NF-κB, a key regulator of pro-inflammatory gene expression, and MAPK, a signaling pathway involved in inflammatory responses, cell proliferation, and stress signaling, while promoting genes involved in hair regeneration. Consistent with these findings, PQFN reduced the expression of key senescence-associated genes, including p16, p21, p53, and TGF-β1, which were reduced by 4.7-, 3.8-, 3.2-, and 5.1-fold, respectively. The reduction of TGF-β1 is particularly relevant in AGA because this senescence-associated secretory factor contributes to the propagation of inflammation and aging throughout the perifollicular microenvironment (56).

Beyond reversing cellular senescence, PQFN-integrated microneedles also modulated pathways directly associated with hair regeneration. Treatment reduced the expression of SRD5A2, a key driver of androgen-mediated follicular miniaturization (57), while increasing VEGF, a major regulator of angiogenesis that supports nutrient and oxygen delivery to growing hair follicles, and β-catenin, a central signaling molecule involved in hair follicle regeneration and anagen initiation (58). These transcriptional changes were reflected in improved vascular remodeling, characterized by increased blood vessel density, diameter, and flow velocity, ultimately promoting the transition of hair follicles from the telogen to the anagen phase. Overall, these findings demonstrate that the gas-propelled PQFN platform works not only as an efficient follicular delivery system but also as a multifunctional therapeutic strategy capable of simultaneously reversing oxidative stress, cellular senescence, and vascular insufficiency, three major pathological drivers of AGA.

Together, these studies suggest that nanoparticle-integrated microneedle platforms can address several alopecia-associated mechanisms, including immune dysregulation, reduced vascularization, and oxidative-stress associated follicular aging. By combining localized delivery with controlled release and improved follicular targeting, these systems may support hair regeneration while reducing systemic exposure. Challenges related to nanoparticle biodistribution, clinical translation, and large-scale manufacturing must still be addressed before these systems can achieve clinical translation (59).

## 4 Nanoliposome-integrated microneedle platforms for alopecia treatment

Nanoliposomes are nanoscale (typically ~50–300 nm) phospholipid vesicles in which one or more concentric lipid bilayers enclose an aqueous core (60). Because they combine a hydrophilic interior with a lipophilic bilayer, they can carry both water-soluble and lipid-soluble drugs, and their surface chemistry, charge, and size can be tuned during fabrication (60). They are most commonly produced by thin-film hydration, ethanol injection, reverse-phase evaporation, or microfluidic mixing, frequently followed by extrusion or homogenization to narrow the size distribution (61). Conventional liposomes incorporate cholesterol to rigidify and stabilize the bilayer, whereas “nanoliposomes” usually denotes the smaller, more uniform vesicles favored for transdermal and follicular delivery. When these vesicles are embedded within a microneedle matrix, they add a second level of formulation control to conventional dissolving microneedles: the therapeutic agent is first loaded into the lipid vesicle, and that vesicle is then embedded within the microneedle (62). This hierarchical design is attractive in alopecia because treatment efficacy depends not only on crossing the stratum corneum, but on sustaining drug exposure within the perifollicular niche over repeated treatment cycles (4, 9).

Conventional polymer-loaded microneedles can improve scalp penetration, but they often release cargo rapidly once the matrix hydrates and dissolves (5, 17). By contrast, nanoliposomes can protect labile payloads, accommodate both hydrophilic and lipophilic molecules, improve retention within follicle-associated lipid environments, and reduce reliance on irritation-prone solvent systems used in standard topical formulations (60). The lipid bilayer can prolong cutaneous residence time, buffer sensitive molecules from premature degradation, and modulate how the released drug partitions between the interfollicular epidermis and the follicular reservoir (60, 63). These features are especially important in alopecia, where the relevant biological targets are not superficial keratinocytes alone, but dermal papilla cells, follicular stem-cell compartments, the perifollicular vasculature, and an inflamed immune microenvironment that requires prolonged local modulation (64).

In the subsections that follow, we examine two representative nanoliposome–microneedle systems in detail: a kopexil/kopyrrol codelivery platform for AGA and a cholesterol-free ginsenoside Rg3 liposome platform; the shared strengths and current limitations of the nanoliposome approach are then considered alongside those of the other platforms in Section 8.

### 4.1 Kopexil/kopyrrol codelivery nanoliposomes

A representative example of nanoliposomes integrated into a microneedle platform is the kopexil/kopyrrol codelivery system described by Zhang et al. (62). Kopexil (diaminopyrimidine oxide) and kopyrrol (pyrrolidinyl diaminopyrimidine oxide) are structural analogs of MXD that are already commercialized and notably less toxic than MXD itself. Individually, they produce weaker hair-regrowth effects than MXD, so co-encapsulating them was used to recover efficacy through synergy while preserving their favorable safety profile. The two actives were co-encapsulated at a 2:1 mass ratio (kopexil:kopyrrol) within nanoliposomes (KK-NLPs) with a particle size of ~123 nm and a ζ-potential of −31.4 mV at a lipid-to-drug molar ratio of 1:2.5, and these vesicles were then embedded within a dissolving HA microneedle array (KK-NLPs@MNs) at a mass ratio of 1:20 (62) (**Figure 3**).

**Figure 3:**
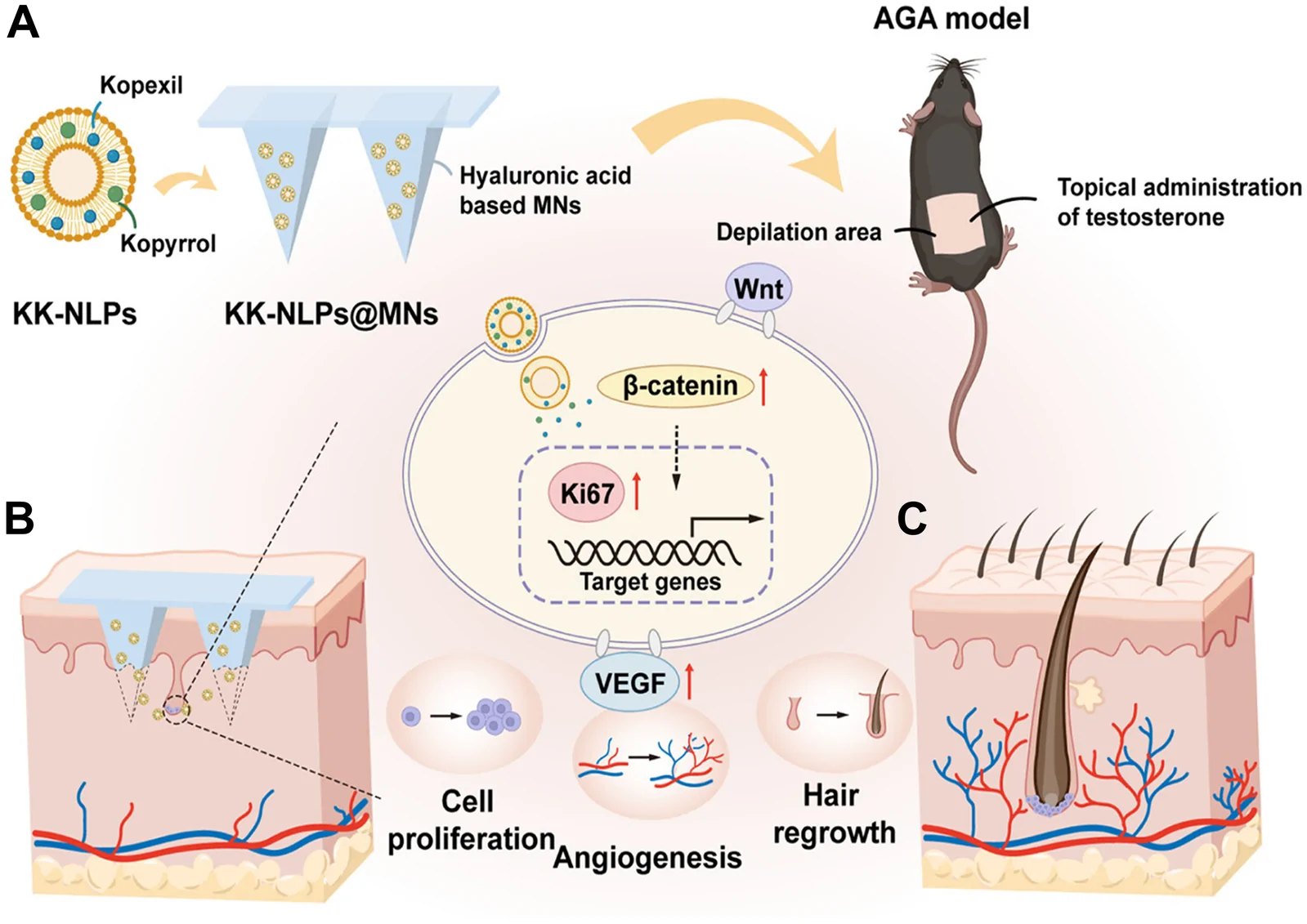
Schematic of a codelivery NLPs-integrated MNs (KK-NLPs@MNs) delivery platform for the treatment of AGA. **(A)** Fabrication of KKNLPs@MNs. Kopexil and kopyrrol are first coencapsulated in NLPs (KK-NLPs) and then loaded into HA-based MNs. A testosterone-induced AGA model is constructed in the C57BL/6 mouse. **(B)** After the MNs are applied to the skin, HA is gradually dissolved and the KK-NLPs are taken up by the target cells. Released from NLPs, kopexil and kopyrrol exert their effects on promoting cell proliferation, angiogenesis, and hair regrowth through regulating the expression of related factors. **(C)** With the multiple actions of KK-NLPs@MNs, the HFs in the telogen accelerate into the anagen and the hair begins to regenerate. Reproduced with permission from Zhang et al., ACS Applied Materials & Interfaces; published by ACS, 2024 (62).

Mechanistically, the platform engages several hair-cycle levers at once. After the HA needles dissolve, the released KK-NLPs are internalized by human dermal papilla cells, where kopexil and kopyrrol act synergistically to upregulate Ki-67 (proliferation), β-catenin (Wnt-pathway activation and anagen induction), VEGF (angiogenesis), and CD31 (endothelial/microvascular marker). These increases were quantified against both untreated control cells and free kopexil/kopyrrol (free KK). The Ki-67-positive rate of dermal papilla cells treated with KK-NLPs@MNs reached ~65%, the highest of any group. Additionally, VEGF secretion was ~36% higher than free KK and ~21% higher than non-embedded KK-NLPs, and cell migration at 24 h was nearly 65%, 39% higher than free KK. The formulation also reduced intracellular reactive oxygen species by >50% relative to the oxidatively damaged control. Together these responses promote dermal papilla cell proliferation and migration, exert antioxidative effects, drive peripheral angiogenesis, and accelerate the transition of hair follicles from telogen into the anagen growth phase.

In a testosterone-induced AGA model in C57BL/6 mice, KK-NLPs@MNs produced hair regeneration comparable to topical MXD while using about 38% of the drug dose and showing lower toxicity than free-drug controls. Encapsulation protected the two actives during fabrication, maintaining their fixed ratio, and facilitated cellular uptake after intradermal delivery. The drove 79.8% of follicles into anagen, compared with 78.5% for MXD and 72.0% for the same actives delivered as non-embedded liposomes (62). These findings support a carrier-mediated codelivery effect, although they do not by themselves establish a long-lived intradermal depot.

This study also illustrates a broader design principle. AGA involves altered perifollicular remodeling, impaired microcirculation, inflammation, and shortened anagen duration. Codelivery liposomal microneedles can engage several of these mechanisms while preserving a defined drug ratio. Whether this strategy is clinically superior to simpler single-agent formulations will require further head-to head testing.

### 4.2 Cholesterol-free ginsenoside Rg3 liposomes

An especially instructive variation on liposome-integrated microneedles is to make the vesicle membrane itself part of the therapeutic design. In the cholesterol-free Rg3 system reported by Yang et al., ginsenoside Rg3 liposomes (Rg3-LPs) were incorporated into a 5:2 w/w composite hyaluronic-acid/Bletilla striata polysaccharide (HA/BSP) dissolving microneedle (65). The liposomes were ~107 nm in diameter and maintained similar encapsulation efficiencies as cholesterol-based analogs. Bletilla striata polysaccharide is a natural polysaccharide extracted from the orchid Bletilla striata; it has intrinsic hemostatic, anti-inflammatory, and hair-growth-stimulating properties and, because of its high viscosity, both stiffens the needles and supports sustained release, complementing the HA matrix (65).

This system is notable because it links carrier design to an alopecia-specific rationale. The authors omitted cholesterol from the liposomal membrane and used Rg3 as a membrane-forming substitute. Their rationale was that cholesterol is a steroid precursor of testosterone, and the testosterone metabolite dihydrotestosterone (DHT) miniaturizes follicles and prolongs telogen, so a cholesterol-laden carrier could in principle feed the very pathway that drives AGA. Rg3 is a structural analog of cholesterol whose C3 hydroxyl group binds the hydrophilic head groups of the phospholipids; this shortens the distance between bilayers, reduces vesicle size, and improves liposome stability while avoiding the cholesterol-to-DHT liability. Rg3 itself also stimulates follicles and upregulates VEGF, so the membrane-forming component doubles as a bioactive agent.

The data support enhanced transdermal delivery rather than a conventional long-term depot. The HA/BSP array was approximately 900 μm high, produced reversible microporation, and dissolved within 30 min; confocal imaging confirmed deeper deposition of fluorescently labeled cargo than free liposomes (**Figure 4A**). Cumulative transdermal delivery reached 83.87% at 72 h for Rg3-MNs versus 14.27% for free Rg3 liposomes (**Figures 4B, C**), indicating substantially improved delivery across the skin barrier.

**Figure 4:**
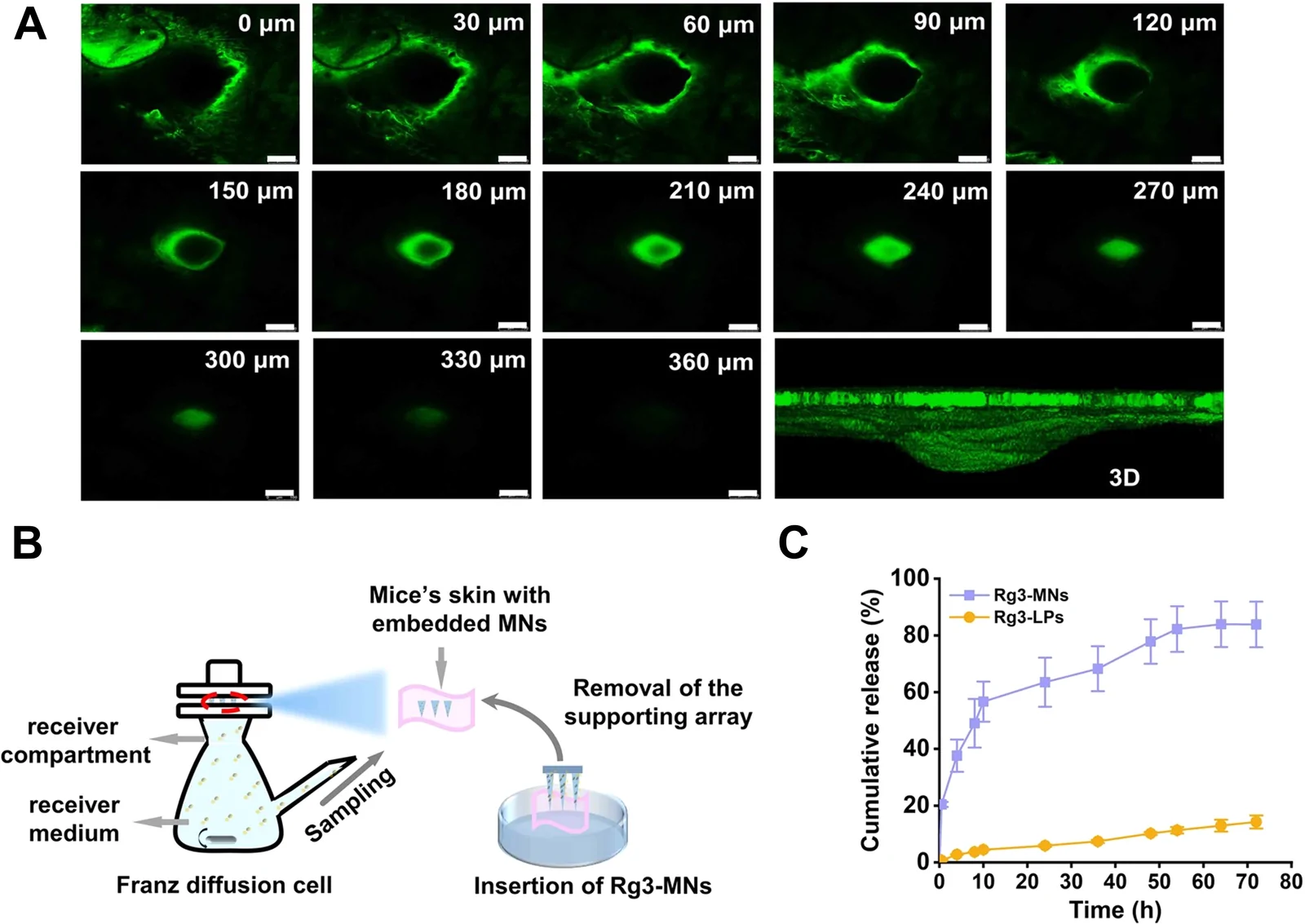
Skin penetration characterization and drug release of the Rg3-MNs. **(A)** CLSM (confocal laser scanning microscopy) images comparing the depth and intensity of fluorescently labeled cargo in C57BL/6 mouse skin after Rg3-MN application versus free liposomes, showing that the microneedles deposit drug in the deeper dermal and perifollicular region rather than the superficial epidermis (scale: 100 μm). **(B)** Schematic diagram of the transdermal drug delivery research. **(C)** In vitro transdermal drug release curve, showing 83.87% cumulative release at 72 h for Rg3-MNs versus 14.27% for free liposomes. Data are presented as mean ± SD (n = 3). Reproduced with permission from Yang et al., Regenerative Biomaterials; published by Oxford Academic, 2024 (65).

In both telogen effluvium (TE) and testosterone-induced AGA mouse models, dosing every 3 days produced hair regrowth comparable to daily topical MXD despite a much lower dosing frequency (once every 3 days versus daily MXD); regenerated hair density in the Rg3-MNs group was in fact higher than in the MXD group, while hair-shaft diameter was comparable to MXD, so the reduced dosing burden did not come at the cost of cosmetic quality. At the molecular level, immunohistochemistry showed that the Rg3-MNs group had the highest expression of VEGF (angiogenesis), CD34 (a follicular stem-cell and neovascular marker), and Ki-67 (proliferation) among the tested groups, whereas all three markers were lowest in the untreated AGA model group. The treatment also upregulated Wnt3a/Wnt10b signaling, with transcriptome sequencing of treated skin revealing 823 upregulated genes and significant enrichment of the Wnt/β-catenin pathway. Because Wnt/β-catenin signaling is a master regulator of the telogen-to-anagen transition and of dermal papilla inductive capacity, its upregulation, together with VEGF-driven perifollicular angiogenesis and CD34/Ki-67-marked progenitor proliferation, provides a mechanistic rationale for the observed regrowth. This reinforces the idea that nanoliposomal microneedles can function as both delivery vehicles and follicular microenvironment modulators.

Taken together, nanoliposome-integrated microneedles are most advantageous when the therapeutic problem requires protection of a labile payload, simultaneous loading of hydrophilic and lipophilic agents, or fixed-ratio co-delivery. The lipid vesicle can improve follicular partitioning and lower the dose or dosing frequency, whereas the microneedle bypasses the stratum corneum. These benefits must be weighed against leakage, oxidation, vesicle fusion, and batch-to-batch variation in size, lamellarity, and encapsulation efficiency; indeed, **Table 1** shows that the high encapsulation efficiency of the isolated kopexil/kopyrrol nanoliposomes decreased after incorporation into the HA microneedles. Moreover, rapid needle dissolution does not necessarily create a durable intradermal depot, and the available animal studies do not establish clinical superiority over simpler single-agent microneedles or optimized topical therapy. Nanoliposomes should therefore be selected when cargo protection or coordinated co-delivery provides a clear therapeutic advantage, rather than as a default means of adding formulation complexity (60–65).

## 5 Porous material-integrated microneedle platforms for alopecia treatment

Porous materials are solids whose internal structure is permeated by an interconnected network of nanoscale or microscale cavities, giving them very high surface area and tunable pore volume. The classes most studied thus far are metal-organic frameworks (MOFs) and mesoporous inorganic particles. MOFs are crystalline coordination polymers built from metal nodes bridged by organic linkers, with highly ordered and chemically tunable pores typically below 5 nm (66). Mesoporous inorganic particles are nanomaterials with uniform pore sizes, typically between 2 and 50 nm, including mesoporous silica and mesoporous iron oxide (67, 68). These are distinct from the polymeric and lipid carriers discussed previously: the framework or scaffold’s porosity, rather than a polymer or bilayer envelope, governs loading and release. Porous-material microneedle systems are attractive because they decouple loading capacity from polymer volume and create opportunities for slow release, stimulus responsiveness, and theranostic integration. In alopecia, most of the microneedle-centered porous literature has focused on MOFs, although mesoporous iron oxide and mesoporous silica systems provide additional design cues that are likely to influence future platforms (69–71).

Hair loss often requires prolonged local exposure, yet rapid-dissolving microneedles usually deliver only a short pulse of drug (5, 17). Porous materials address this by acting as intradermal depots after insertion: the drug adsorbs onto the vast internal pore surface and diffuses out gradually, so release is governed by desorption and pore tortuosity rather than by rapid matrix dissolution, enabling slow release of MXD or immunomodulators (e.g., locally delivered corticosteroids, JAK-pathway inhibitors, calcineurin inhibitors) over days to weeks. In addition, the chemistry of MOFs and mesoporous silicas permits rational tuning of cargo binding, pore architecture, and stimulus responsiveness, including responsiveness to pH and local ion concentrations (66, 68). This is relevant to autoimmune AA because the target follicular niche is inflamed rather than chemically static: immune-cell infiltration, cytokine signaling, vascular leakage, and high metabolic activity may create a mildly acidic interstitial microenvironment and alter local ion availability. Acid-labile frameworks such as ZIF-8 could exploit this shift because protonation of imidazolate linkers weakens metal-ligand coordination and accelerates framework breakdown and cargo release under acidic conditions. Ionic conditions are similarly useful as a trigger, because competing ions in interstitial fluid may screen electrostatic drug-carrier interactions or drive ligand/ion exchange (70, 72–74).

### 5.1 UiO-66 porous depots for sustained release and anti-inflammatory hair regeneration

A central example is the UiO-66-based system reported by Zhang et al. for AA (71). UiO-66 is a zirconium-based MOF composed of zirconium clusters bridged by terephthalate linkers; its high surface area, ordered pore structure, slow *in vivo* degradation, and antibacterial activity make it well suited for a microneedle depot. In this study, the drug-loaded carrier was prepared by *in situ* encapsulation. UiO-66 NPs were synthesized by dissolving ZrCl_4_ in acetic acid, combining it with equimolar 1,4-dicarboxybenzene and MXD, and reacting the mixture at 100 °C for 24 h. The resulting MXD@UiO-66 retained the UiO-66 porous structure, with a specific surface area of 1187.7 m²/g, pore volume of 0.46 cm^3^/g, and a pore size of approximately 1.80 nm. The authors attributed drug retention to π–π stacking and electrostatic interactions between MXD and the UiO-66 framework.

The final device was a layered polylactic acid (PLA)/HA microneedle patch, in which MXD@UiO-66 was dispersed in a 10 wt% HA needle-forming phase over a PLA/HA transition layer and a pure-PLA backing. Four formulations (MXD@UiO-66-MNs-0.1, -0.5, -1, and -2) were prepared at 0.1, 0.5, 1, and 2 wt% MXD, respectively.

In an ex vivo rabbit-skin diffusion setup, MXD@UiO-66-MNs released MXD continuously for 28 days. The fraction released ranged from 17% to87% and increased with formulation loading; release was still measurable between days 21 and 28. These findings support the interpretation that the rapidly dissolving HA tips deposited MXD@UiO-66 in the skin, after which slow UiO-66 degradation sustained MXD release.

In an alopecia-induced mouse model, the two MXD-containing patches showed visibly faster hair growth by day 7; however, the lead formulation separated decisively in the second phase, where MXD@UiO-66-MNs-1 was the only group to sustain robust regrowth as the control and all other patch groups reverted to a hairless state, a difference the authors attributed to UiO-66’s slow release of MXD over roughly 28 days versus the immediate metabolism of unprotected drug. Day-28 histology confirmed this advantage and, importantly, framed it in regenerative and anti-inflammatory rather than purely pharmacokinetic terms. Among all groups, MXD@UiO-66-MNs-1 produced the highest hair-follicle density (50.4 follicles/mm², versus only 28.8–32.8 follicles/mm² for the MXD-MNs-1, bare UiO-66-MNs-1, and blank PLA/HA-MNs groups) and the thickest dermis (both established histological markers of follicles re-entering and being maintained in the anagen growth phase). The authors then connected this regrowth to a specific immunological mechanism. Because NF-κB is a central inflammation- and autoimmunity-associated signaling pathway that is aberrantly activated in this alopecia model, they measured its activity directly: MXD@UiO-66-MNs-1 most strongly suppressed phosphorylation of the NF-κB subunit p65, lowering the p-P65/P65 ratio to 0.30, compared with 0.42 for bare UiO-66-MNs-1, 0.48 for MXD-MNs-1, and 0.56 for the blank patch. The sustained local release of MXD from the porous depot therefore did more than prolong drug exposure; it was associated with durable, quantifiable hair regeneration while damping the inflammatory signaling that holds follicles out of anagen, which is precisely the combined immunomodulatory-and-regenerative outcome that motivates encapsulation-integrated microneedles for alopecia.

More broadly, this study illustrates the modularity of porous-carrier microneedles. The HA tips address barrier penetration and dissolve after a short application, the PLA backing supports insertion and removal, and the UiO-66 phase provides the longer-term reservoir. This arrangement couples a brief surface-wear time with extended intradermal release. By sustaining MXD release for up to 28 days in the ex vivo model, the depot may support prolonged pharmacologic activity; however, *in vivo* residence and clearance of the carrier were not quantified. Applying the same modular logic to immunomodulators for AA remains a promising but untested extension.

### 5.2 pH-responsive licorzinc MOFs and theranostic treatment of AA

A more elaborate example is the dual-continuous microneedle patch reported by Ruan et al., which combined pH-sensitive licorzinc MOFs (GA@ZIF-8) with a Zn^2+^-responsive hydrogel sensor (70). The rationale for the pH-responsive MOF is twofold. First, the glycyrrhizic acid (GA)-based licorzinc formulation is a useful immunomodulator but suffers from poor water solubility, a high required oral dose, and the risk of zinc accumulation with long-term systemic use. Adsorbing GA into zeolitic imidazolate framework-8 (ZIF-8, a zinc-imidazolate MOF) markedly improved its solubility at several loadings, with 5:1 found to be optimal. Second, ZIF-8 is acid-labile: it is stable at physiological pH but degrades under the mildly acidic conditions characteristic of inflamed tissue, so the framework acts simultaneously as a solubility-enhancing reservoir and as an acid-triggered release switch that concentrates drug release where inflammation is greatest. The drug-delivery needles were fabricated from HA (10:3 w/w to GA@ZIF-8) and the separate sensing needles were double-cross-linked from N-acryloyl-glycinamide and α-methacrylic acid (10:1 was optimal) and hybridized with a europium(III)–terpyridine (Eu3+–TPy) complex.

Therapeutically, in the cyclophosphamide-induced alopecia model used by the authors, the GA@ZIF-8 delivery unit dissolved within 2 min and promoted hair-follicle recovery. Treatment reduced CD8^+^NKG2D^+^ T-cell infiltration, lowered IL-15 and IFN-γ, and reduced JAK/STAT-associated signaling. These findings are consistent with suppression of the IFN-γ/IL-15 inflammatory loop implicated in AA. The second microneedle unit functioned as a Zn^2+^-responsive sensor. After insertion, the hydrogel needles absorbed interstitial fluid and produced a fluorescence shift as Zn^2+^ replaced Eu^3+^ from the terpyridine complex. The reported detection range spanned 10^-^^8^ to 10^-^¹ M with limited interference from common physiological ions. The sensing arm demonstrated Zn^2+^ detection, but the study did not establish a validated quantitative link between sensor output, disease severity, and drug release *in vivo*.

### 5.3 Additional porous-carrier microneedle systems for alopecia

Although direct alopecia microneedle studies using porous carriers remain relatively few, the available examples are useful in understanding the potential for porosity to be used as a multifaceted design tool. In these systems, pores increase the amount of drug that can be carried, create binding sites that slow diffusion, expose inorganic surfaces or metal ions with their own biological activity, and, in some designs, convert an external or local tissue stimulus into a change in release rate.

A first design uses porosity mainly as a high-capacity, externally actuated reservoir: in the composite microneedle reported by Fang et al., mesoporous iron oxide nanoraspberry particles were combined with poly(vinyl alcohol) (PVA) and fabricated by a 3D-printing/molding method (69), using porosity primarily for hydrophobic drug loading and magnetic/thermal release control. The iron oxide particles were assembled as raspberry-like mesoporous structures; the shorter-reaction MIO-5h particles were approximately 150 nm in diameter and had a surface area of about 265 m²/g, which the authors linked to efficient loading of hydrophobic MXD through pore confinement, oleylamine-modified hydrophobic surfaces, and capillary filling during solvent evaporation. Without an external magnetic field, only about 20% of the MXD was released over 120 min, reflecting relatively strong adsorption within the porous carrier. However, when a magnetic field was applied for 1 min at the start of the experiment, ~23% of MXD was released within 10 min and more than 40% was released by 120 min. Mechanistically, the magnetic field induces Brownian and Néel relaxation losses in the iron oxide domains, generating heat that increases molecular motion and helps overcome drug-particle interactions. *In vivo*, this translated into both pharmacologic and physical effects: Mx-MIOs@MNs produced ~25% and ~40% hair regrowth at days 3 and 7, respectively, and the magnetic-field group achieved an eight-fold higher hair density than untreated controls by day 10. Thus, the porous carrier did three things at once: it loaded MXD, slowed passive release, and enabled on-demand release plus local heating, which can increase cutaneous blood flow, vasodilation, follicular opening, and drug penetration.

A second design instead exploits porosity for multi-agent codelivery and bioactive-ion release. In the quercetin-loaded Zn/Cu dual-doped mesoporous silica nanocomposite microneedle patch (ZCQ/MN) reported by Zhang et al., the porous framework delivers several pathophysiological levers of AGA at once (74). The Zn/Cu-doped mesoporous silica NPs (ZC-MSN) provided mesoporous channels and a high internal surface for quercetin (Qu) loading, while the doped framework served as a controlled source of Zn^2+^ and Cu^2+^. The cumulative 14-day release of Zn^2+^, Cu^2+^, and silicate ions from ZC-MSN reached 4.87, 5.22, and 80.53 µg/mL, respectively. Doping also improved Qu loading: ZC-MSN loaded ~241 mg/g Qu, compared with ~81 mg/g for undoped MSN, largely because Cu^2+^ can chelate Qu. In the microneedle format, ZCQ/MN released more Cu^2+^ over 24 h than ZC/MN, while releasing a similar amount of Qu to directly loaded Qu/MN. The biological result was substantially stronger than either cargo class alone, and the reason is mechanistic rather than simply additive: the three cargos act on complementary arms of AGA pathophysiology at the same time rather than hitting a single target. In a testosterone-induced AGA mouse model, ZCQ/MN reached >95% hair coverage by day 14, with >5.57 cm² of hair-covered area, compared with ~3.38 cm² for Qu/MN and ~2.09 cm² for ZC/MN.

The mechanism in the ZCQ/MN study is particularly important because it shows how porous silica can be used as a systems-level microenvironment modulator. Zn^2+^ and Cu^2+^ can inhibit 5α-reductase, reducing the conversion of testosterone to DHT, but the authors found that the triple combination was more effective than single or dual components. In DHT-injured human hair follicle dermal papilla cells, ZCQ treatment upregulated anagen-associated genes (MMP-2, PDGF, c-Myc, and CCN1) and downregulated TGF-β1, a catagen-associated signal. RNA sequencing showed 722 genes upregulated and 1458 genes downregulated after ZCQ treatment, with pathway changes involving steroid biosynthesis, TNF signaling, TGF-β signaling, VEGF/HIF-1 angiogenesis, and AMPK-related skin regeneration. Quercetin contributed anti-inflammatory chelating antioxidant activity, reducing macrophage TNF-α and IL-6 signaling, while also chelating Cu^2+^, which likely helped suppress the pro-inflammatory tendency of free Cu^2+^ while preserving its pro-angiogenic contribution. Stem-cell activation was reflected by increased hair-follicle precursor formation, CK19-positive follicular structures, and maturation of hair shafts. Relative to the controls, this maturation was striking: in the ZCQ/MN group almost all follicles had grown pigmented, fusiform (mature) hair shafts—comparable to healthy, non-AGA skin—whereas the Qu/MN and ZC/MN groups produced only a few pigmented shafts that remained rounded and immature, and the blank-gauze and unloaded-microneedle (MN) groups stayed largely in the primary, pre-growth stage. Within this comparison, Qu appeared especially important for differentiation and the Zn/Cu combination for migration.

A third design ties porosity to a stimulus-responsive, recyclable platform: Aminov et al. extended the porous-carrier concept into a 3D-printed dissolving microneedle system with a recyclable polymerizable deep eutectic solvent (PDES) base and detachable, pH-responsive tips loaded with MXD-encapsulated ZIF-8 NPs (75). In this case, porosity is tied to pH-responsive retention and release. ZIF-8 was loaded with MXD after APTES modification, producing an encapsulation efficiency of nearly 90% and a drug-loading efficiency of 9.1%. Each 6 × 6 microneedle patch contained approximately 122 µg MXD, or about 3.39 µg per needle. The release profile was strongly pH dependent. At pH 5.0, approximately 80% of encapsulated MXD was released within 24 h, whereas 58% was released at pH 6.5 and essentially no MXD was released at pH 7.0. Zn^2+^ release showed the same acid sensitivity: after approximately 53 h, cumulative Zn^2+^ release reached ~78% at pH 5.0, ~45% at pH 6.5, and only ~5% at pH 7.0. Mechanistically, acidic conditions protonate the imidazolate linkers and weaken Zn-imidazolate coordination, destabilizing the porous ZIF-8 framework and liberating both the MXD and Zn^2+^. *In vivo*, MXD-ZIF-8 MNs produced the densest and most uniform hair coverage by day 26 in a cyclophosphamide-induced alopecia model, with H&E showing deeper and more numerous follicles than MXD alone, ZIF-8 MNs, blank MNs, or untreated controls. Ki-67 and CD31 staining also increased at 24 and 72 h, consistent with enhanced perifollicular proliferation and angiogenesis. This design uses the porosity of the carrier as a tunable switch, while the reusable PDES base separates mechanical support from the disposable drug-loaded tip.

Several broader takeaways follow from these three systems. First, porosity can solve different problems depending on the carrier: the mesoporous iron oxide emphasizes high-surface-area loading plus remote magnetic actuation; the mesoporous silica demonstrates multi-cargo loading, ion release, and surface-mediated chelation; and the ZIF-8 allows for framework-protected cargo storage with acid-triggered release. Second, the porous phase can carry biological meaning beyond drug storage. Released Zn^2+^ and Cu^2+^ can regulate DHT-related enzymes, endothelial activation, angiogenesis, and hair-follicle cell behavior, while Qu modulates oxidative and inflammatory signaling. Third, the best systems use microneedles and porous particles for complementary jobs: the microneedle breaches the stratum corneum and localizes the material near the follicle, while the porous phase controls residence time, release trigger, and biological microenvironment modulation.

Porous carriers offer several advantages: their high internal surface area can support substantial loading, long-lived depots, multi-agent delivery, and pH-, ion-, or magnetically triggered release, making them attractive when sustained or disease-responsive exposure is therapeutically necessary. However, their limitations are equally evident. Multi-step synthesis, residual solvents or reagents, particle and pore heterogeneity, and sensitivity of release to local pH or ionic conditions can impair scale-up and reproducibility. In addition, prolonged retention of zirconium-, zinc-, iron-, or silicon-containing materials raises unresolved questions about biodegradation, clearance, cumulative inflammation, and potential metal-ion toxicity during chronic scalp treatment; ex vivo release profiles may not predict these processes *in vivo*. Porous systems are therefore most defensible for payloads that genuinely require high loading, a prolonged depot, a trigger, or multi-component release, provided that carrier fate and trigger reliability are demonstrated in disease-relevant models (66–75).

## 6 Microsphere-integrated microneedle platforms for alopecia treatment

Microsphere-based microneedle systems use biodegradable polymeric particles, commonly referred to as microspheres (MSs), as localized drug reservoirs within the skin. MSs are commonly fabricated from polymeric materials, including synthetic polymers such as PLGA, PLA, and PCL, as well as natural polymers such as chitosan, alginate, and gelatin (76–78), and can encapsulate different therapeutic agents ranging from small-molecule drugs to bioactive molecules such as peptides, proteins, and nucleic acids (76, 79, 80). They are typically prepared using techniques such as emulsion–solvent evaporation, spray drying, or coacervation, depending on the polymer, payload, and desired release profile (81). In MN platforms, MSs extend the role of dissolving needles beyond rapid payload deposition by introducing a secondary sustained-release reservoir. After insertion, the dissolving needles cross the outer skin barrier and deposit the drug-loaded MSs within the skin (76, 78). The deposited MSs remain locally within the treated region and function as local drug depots, gradually degrading and releasing the encapsulated agent over time. This drug-depot mechanism is shown in **Figure 5A** through the MXD-loaded PLGA MSs system described below.

**Figure 5:**
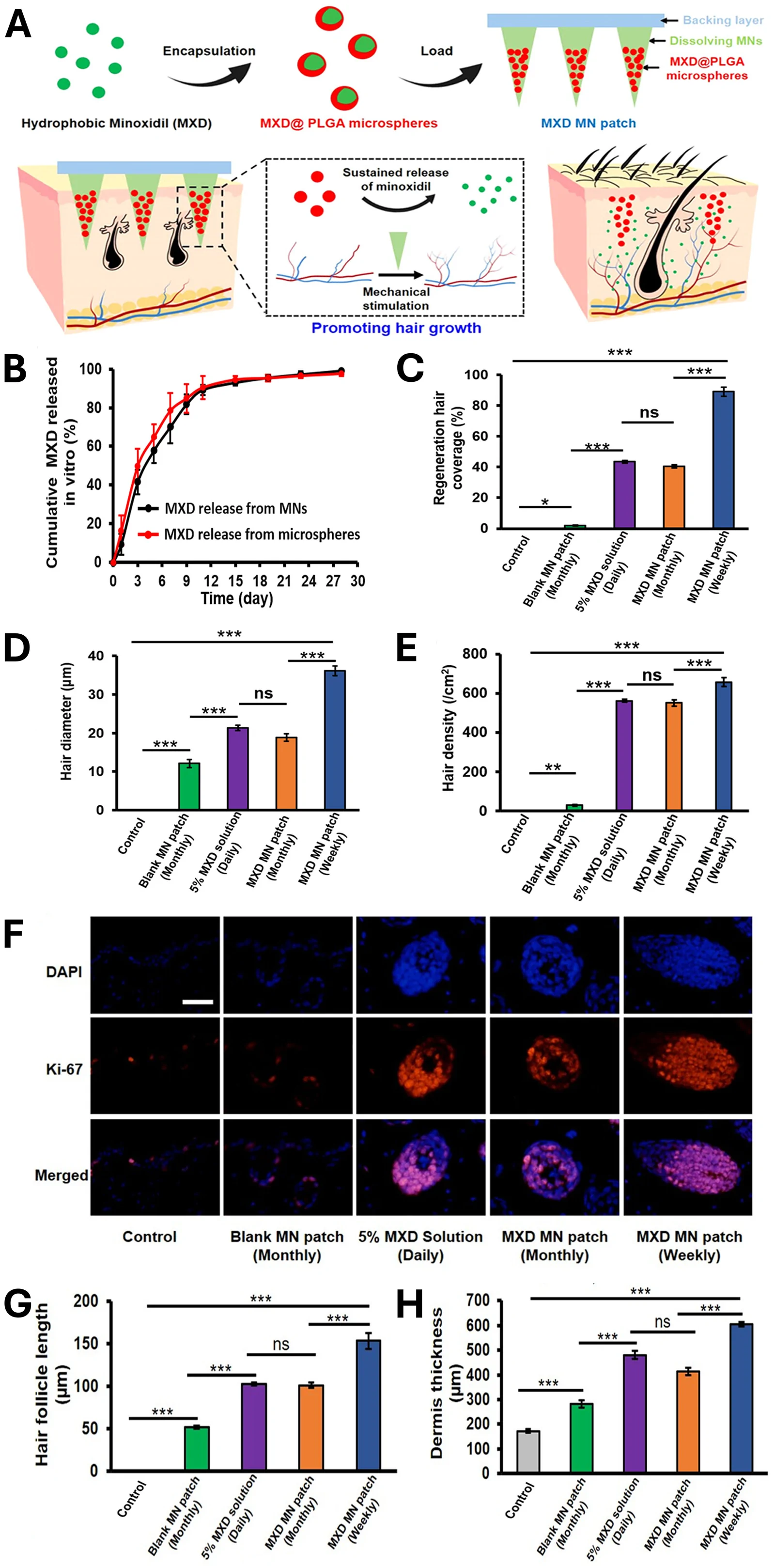
MXD-loaded MSs microneedle patch for long-acting hair regrowth therapy. **(A)** Schematic representation of MXD encapsulation into PLGA MSs, loading into dissolving PVA/sucrose microneedles, and intradermal deposition after patch application. **(B)** In vitro cumulative MXD release from the MXD-loaded MN patch and isolated PLGA MSs. **(C–E)** Quantification of hair regeneration coverage, hair diameter, and hair density in untreated controls, blank MN patches, daily topical 5% MXD solution, monthly MXD MN patches, and weekly MXD MN patches. **(F)** Representative Ki-67 immunofluorescence staining of hair follicles after treatment. **(G, H)** Quantification of hair follicle length and dermis thickness. Reproduced with permission from Yin et al., ACS Applied Materials & Interfaces; published by ACS, 2023 (87).

This depot-based strategy has been adapted to target different biological drivers of alopecia. In AGA, one example used a dissolving MN patch loaded with dutasteride (DUT)-encapsulated PLGA MSs (21), referred to as DUT-MS-MNs. DUT inhibits 5α-reductase, reducing the local conversion of testosterone into DHT, a key androgen that progressively reduces follicle size, leading to thinner hairs and a shorter active growth phase in AGA (21). To enable this localized and sustained DUT delivery, the drug was formulated into PLGA MSs with an average size of 9.8 ± 2.8 μm, with DUT accounting for 14.3 ± 0.7% of the final MSs mass and 86.1 ± 4.2% of the initially added drug being successfully encapsulated. These MSs were embedded in an HA/PVP tip matrix and delivered through a dissolving MN array, with a patch drug content of 51.2 ± 5.54 μg. The DUT-MS-MN formulation also showed acceptable cytocompatibility, with cell viability remaining above 80% across the tested formulations and concentrations. *In vitro*, DUT-MS released only ~9.6% of the drug during the first 12 h, followed by a gradual increase to ~90.8% over approximately 20 days, confirming sustained drug release. In the testosterone-induced AGA mouse model, daily and weekly DUT-MS-MNs administration resulted in approximately 85% and 80% regrowth hair coverage, respectively, by day 22. The comparable response between weekly and daily treatment is consistent with the depot function of the PLGA MSs, which likely maintained local DUT exposure between applications and sustained inhibition of DHT formation despite the reduced dosing frequency (82, 83). Histological examination showed enlarged, melanin-containing follicles in the daily and weekly DUT-MS-MNs groups, consistent with their transition into the active growth phase, whereas follicles in the testosterone-treated model remained superficial and atrophic. These two DUT-MS-MNs groups also showed improved dermal thickness and follicular development relative to the model group. At the biochemical level, the relative fluorescence intensity of transforming growth factor beta 1 (TGF-β1) decreased from approximately 93 in the testosterone-treated model group to ~60 and ~55 following daily and weekly DUT-MS-MNs administration, respectively. Intradermal DHT content also decreased from ~1.34 nmol/g in the model group to ~0.92 nmol/g with daily treatment and ~0.98 nmol/g with weekly treatment. Mechanistically, this reduction in local DHT may have lowered androgen receptor-associated catagenic signaling within the follicular niche, including TGF-β1-linked regression pathways, thereby supporting follicular enlargement, pigmentation, and dermal recovery (84–86). These biochemical changes complement the histological recovery observed after treatment. The DUT-MS-MN patch was also well tolerated by the skin at the application site. Following removal of the patch after a single 10-min application, the micropores began to close within 5 min and were no longer visible after 20 min.

A similar PLGA MSs-based approach was used to achieve sustained local release of MXD (87). Whereas the dutasteride platform was used to modulate androgen-associated signals, the MXD-based system was designed to sustain local follicular stimulation through improved intradermal delivery. **Figure 5A** summarizes this design: hydrophobic MXD is first encapsulated into PLGA MSs, which are then loaded into a dissolving PVA/sucrose microneedle patch for deposition within the skin after application. The MSs were prepared using 150 mg of MXD and 180 mg of PLGA, corresponding to an initial MXD: PLGA mass ratio of ~0.83:1. They showed an average diameter of 15.3 ± 2.2 μm, with 13.6 ± 3.2% of the initially added MXD being encapsulated within the MSs. The final patch contained 56.3 ± 9.2 μg of MXD per patch. *In vitro* release testing showed that the MXD formulation supported sustained release with almost no initial burst. As shown in **Figure 5B**, approximately 90% of MXD was released within 14 days, with complete release occurring at around 1 month, consistent with the gradual degradation of the PLGA MSs. The patch also showed efficient ex vivo skin delivery: approximately 98.0% of the microneedles formed detectable insertion sites, while 94.5 ± 2.1% of the fluorescent model payload initially contained in the patch was transferred into the skin after 20 min. This efficient intradermal deposition may help explain why a much lower nominal MXD dose could still produce biological effects comparable to topical treatment, since the MSs placed the drug closer to the follicle-containing dermal compartment (87, 88). *In vitro*, hemolysis ratios remained close to zero after incubation with two or four patches (0.12 ± 0.43% and 0.18 ± 0.37%, respectively), indicating minimal red blood cell damage and supporting the hemocompatibility of the patch materials.

In the AGA mouse model, the monthly MXD microneedle patch produced a response similar to the daily 5% topical MXD formulation, as shown in **Figures 5C–E**: both groups reached approximately 40% hair regrowth coverage and about 580 hairs/cm² in hair density, although the topical MXD group showed a slightly higher hair diameter (~22 μm) than the monthly patch (~18 μm). By contrast, once-weekly administration of the MXD MN patch produced a stronger response by day 42, increasing hair coverage to ~90%, hair diameter to ~35 μm, and hair density to ~650 hairs/cm². Notably, this response was achieved with a per-patch MXD dose that was approximately 890-fold lower than the 50 mg dose used in the topical MXD formulation (87). The stronger weekly response may reflect the combined action of intradermal MS depots and repeated MN-induced tissue repair. By maintaining MXD within the perifollicular compartment, the sustained-release depots may have prolonged drug exposure, while repeated microinjury may have supplied regenerative wound-healing cues, potentially involving Wnt/β-catenin signaling, that enhanced follicular responsiveness to treatment (87, 89–91). Histological and immunofluorescence analyses further supported the regenerative response. **Figure 5F** shows minimal Ki-67 immunofluorescence in the control group, whereas the weekly MXD patch group displayed a visibly stronger and more widespread signal. Because Ki-67 is a marker of actively proliferating cells, this finding indicates greater proliferative activity within the hair follicles. Consistently, **Figures 5G, H** show that once-weekly administration of the MXD MN patch resulted in a follicle length of ~150 μm and a dermal thickness of ~600 μm. Both measurements were higher than those obtained with daily topical MXD (~100 μm and ~490 μm, respectively) and once-monthly MXD MN patch administration (~100 μm and ~440 μm, respectively), further supporting the stronger regenerative response observed with the weekly treatment schedule (87). Together, the increased Ki- 67 signal, longer follicles, and greater dermal thickness are consistent with a stronger shift toward anagen (92, 93). The MXD-loaded PLGA MSs MN patch was also well tolerated *in vivo*, with no evident inflammation or tissue damage at the treatment site. Taken together, the DUT and MXD studies show that MS-based MN systems can function as local drug depots for alopecia therapy while producing drug-specific biological outcomes. DUT-MS-MNs primarily reduced androgen-associated signals such as DHT and TGF-β1, whereas MXD-loaded MS-MNs improved follicular stimulation, hair coverage, hair diameter, hair density, follicle length, and dermal thickness (21, 87). At present, these systems provide a preclinical strategy for sustaining local drug exposure and reducing treatment frequency in alopecia. However, drug-loading capacity, patch size, formulation scalability, and long-term pharmacokinetic and safety profiles require further optimization to translate these benefits into a practical treatment for human use.

## 7 Emerging system for alopecia treatment: light-guiding microneedle systems

While microneedles were initially designed for drug delivery, newer platforms now use them to deliver physical stimuli, such as light, directly to target tissues, enabling innovative, minimally invasive treatments for AGA.

The treatment options for AGA have seen little innovation over the past decades, remaining largely limited to topical MXD and oral finasteride (11). Both remain therapeutic pillars, although each has well-recognized limitations. MXD can cause local adverse effects, including scalp pruritus, irritation, contact dermatitis, erythema, and other cutaneous problems, which can reduce treatment adherence (94). Finasteride, however, has been associated with concerns regarding sexual adverse effects, including decreased libido, erectile dysfunction, and ejaculatory changes. These risks have been evaluated in systematic reviews and continue to be monitored by regulatory agencies (95).

Low-level light therapy (LLLT), also known as photobiomodulation (PBM), is a non-invasive approach that uses low-intensity red or near-infrared light to stimulate cellular activity without thermal tissue damage (96). In AGA, red-light LLLT has improved hair density and hair-shaft thickness in selected patients, making it a potential adjunctive option. Proposed mechanisms include absorption by cytochrome c oxidase, increased ATP production, modulation of ROS, and nitric oxide (NO) release. Together, these effects may enhance cellular metabolism, reduce inflammation, improve blood, and support anagen maintenance (97, 98).

The therapeutic effects of LLLT depend strongly on the wavelength used, as different wavelengths interact with biological tissues in distinct ways. LLLT has been investigated across the visible and near-infrared spectrum, including blue (400–470 nm), green (495–570 nm), yellow (570–590 nm), red (630–700 nm), and near-infrared light (700–1200 nm). Shorter wavelengths are more efficiently absorbed by endogenous chromophores, notably melanin and hemoglobin, and are subject to increased scattering within the skin. In contrast, longer wavelengths undergo less absorption and scattering, allowing photons to penetrate deeper into biological tissues (99) (**Figure 6A**).

**Figure 6:**
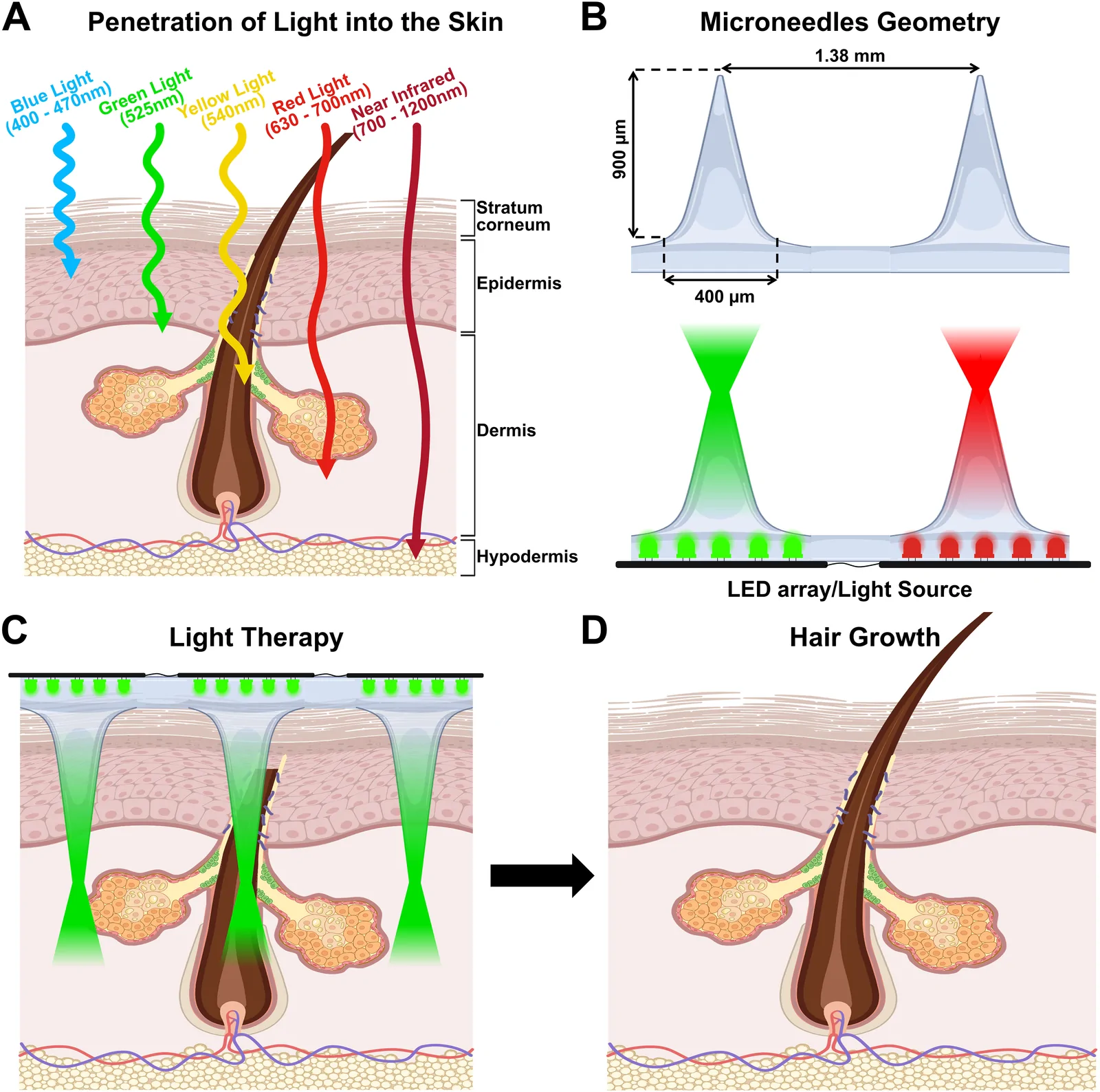
**(A)** Approximate penetration depths of photobiomodulation wavelengths in skin. **(B)** Geometry and light-guiding principle of the transparent microneedle array (102) **(C)** Schematic representation of green light delivery to deep hair follicles using light-guiding microneedles. **(D)** Conceptual hair-growth outcome following treatment.

Red light (630–700 nm) is the most studied wavelength for AGA and is generally described as penetrating approximately 2–5 mm into skin, potentially reaching the dermal papilla and other deeper follicular structures (40). Green light (495–570 nm) penetrates more shallowly, approximately 0.5–1 mm, because it is more strongly absorbed and scattered in superficial tissue (41). Although this limits convential green-light delivery to deep follicles, green light has been associated with tissue repair, fibroblast activity, and angiogenic responses (100, 101).

A recent study introduced a light-guiding microneedle patch (LMNP) fabricated from transparent PMMA by photopolymerization. PMMA was selected for its refractive index (approximately 1.49), which exceeds that of skin (approximately 1.4). This difference supports total internal reflection within the microneedles and enables light transmission from the base toward the tip increasing local light delivery near hair follicles (102).

The curved patch was designed to conform to the scalp and incorporated pyramidal microneedles with convex faces. It contained 105 needles/cm², with an average height of 900 micrometers, a base width of 400 micrometers, a needle spacing of 1.38 millimeters. Each patch covered an area of 21.7 cm² (**Figure 6B****).** The array was mounted on a flexible textile substrate, which enabled it to follow the curvature of the frontal scalp (102).

For phototherapy, the LMNP was combined with a portable light-emitting diode (LED) helmet that emitted red light at 633 nm and green light at 522 nm (**Figures 6C, D**). The helmet was placed at 10 mm above the scalp and operated at 50 mW/cm². During treatment, a microneedle patch was placed on the frontal scalp and secured with a transparent elastic band to ensure proper contact and penetration depth. The right side of the scalp was exposed to green light, while the left side was exposed to red light, allowing a direct comparison of the biological responses to each wavelength within the same individual under consistent conditions (102).

Following a six-month evaluation period, the investigators observed a substantial increase in terminal (non-vellus) hair density in both treatment groups. Specifically, in the red LED group, hair density rose from 46.66 ± 14.91 hairs/cm² at baseline to 68.59 ± 20.12 hairs/cm². The beneficial effects of red light are potentially linked to the activation of biological pathways that promote hair follicle growth. Although these pathways were not explicitly examined in the clinical LMNP study, another study demonstrated that exposure to 655-nm red LED light significantly increased the proliferation of hair matrix keratinocytes, as evidenced by an increase in Ki-67-positive cells (cells expressing the nuclear proliferation marker Ki-67) from 24.7% to 62.5% (103). Concurrently, immunofluorescence analysis revealed an elevated expression of β-catenin (the central intracellular mediator of the canonical Wnt signaling pathway), LEF1 (the transcription factor responsible for activating Wnt-responsive genes involved in hair follicle development), and phosphorylated GSK3β (p-GSK3β) (the inactive form of GSK3β that inhibits β-catenin degradation and thereby enhances Wnt signaling). The mean fluorescence intensities, expressed as relative optical density increased from approximately 0.032 to 0.068 for β-catenin, from 0.023 to 0.043 for LEF1, and from 0.022 to 0.036 for p-GSK3β. Collectively, these molecular alterations suggest activation of the Wnt/β-catenin signaling pathway, which facilitates hair follicle activation, prolongs the anagen phase, and stimulates proliferation of hair matrix cells (103).

Similarly, terminal hair density in the green LED group increased from 49.40 ± 19.91 hairs/cm² to 78.64 ± 19.11 hairs/cm². Although the numerical increase was larger than in the red-light group, the between-group difference was not statistically significant. The authors proposed that green light may support hair growth through wound-healing, fibroblast, and angiogenic responses. Vellus hair density remained statistically unchanged in both groups, suggesting that the gain in coverage reflected thickening of existing terminal hairs rather than an increase in vellus hairs.

Both treatment groups showed a significant increase in hair-shaft diameter. Mean diameter increased from 79.62 ± 8.13 µm to 87.29 ± 6.02 µm in the red LED group and from 80.13 ± 8.65 µm to 90.39 ± 6.56 µm in the green LED group. The difference between red and green LED groups was not statistically significant (104, 105).

This improvement aligns with reversal of follicular miniaturization, a hallmark of AGA characterized by progressive shrinkage of terminal hair follicles and the production of thinner, shorter vellus-like hairs. Unlike traditional low-level light therapy, which is limited by tissue scattering and absorption that reduce light reaching deeper follicles, the LMNP acts as an optical waveguide. It directs light toward the target tissue, potentially improving treatment and reducing energy loss. These findings underscore the potential of light-guiding microneedles as minimally invasive platforms for localized phototherapy, thereby extending their applications beyond conventional drug delivery. However, the study did not include control groups receiving LED irradiation alone or microneedling alone; consequently, the individual contributions of each component to the observed therapeutic effects could not be ascertained. Furthermore, light-guiding microneedles face challenges such as tissue absorption and scattering, as well as interpatient variability in scalp thickness, pigmentation, and hair density. Scalp curvature and hair can also affect the consistency of insertion and light distribution. Microneedle deformation and manufacturing complexities could also reduce optical efficiency and hinder large-scale production.

**Table 1** provides a comparative overview of the microneedle platforms reviewed for alopecia treatment, highlighting their delivery systems, encapsulation strategies, therapeutic agents, alopecia subtypes, mechanisms of action, drug-loading capacities, release profiles, and advantages over conventional microneedle designs. Collectively, these platforms illustrate how advances in encapsulation and controlled drug delivery can improve therapeutic efficacy by enhancing follicular targeting, prolonging local drug activity, modulating inflammatory and immune-related processes, and promoting hair follicle regeneration.

## 8 Current challenges and future directions in encapsulation-integrated microneedle therapies for alopecia

The central challenge in alopecia microneedle research is no longer simply whether increasingly complex platforms can be built, but which designs are biologically justified, manufacturable at scale, stable during storage, safe for chronic scalp use, and competitive with current standards of care. This question is especially important now that AA has FDA-approved systemic therapies: baricitinib became the first FDA-approved systemic treatment for severe AA in 2022 (106), followed by ritlecitinib in 2023 and deuruxolitinib in 2024 (107, 108). Localized microneedle platforms must therefore demonstrate a clear benefit alongside these options—for example, lower systemic exposure, less frequent dosing, or efficacy in a clinically defined subgroup.

However, this does not diminish the value of localized delivery. A successful alopecia microneedle platform should ideally reduce total systemic exposure, lower chronic dosing burden, improve local pharmacokinetics at the follicle, and address mechanisms that current oral therapies do not solve well, such as poor follicular drug retention, inadequate local remodeling, or the need for localized combinatorial therapy. The future of the field depends on making those advantages explicit (109–111). The subsections that follow develop this case in turn: matching encapsulation strategy to the immunological and regenerative biology of each alopecia subtype (Section 8.1), confronting the manufacturing, stability, and safety barriers that complex carriers introduce (Sections 8.2–8.3), and outlining what a credible path to clinical translation and the next generation of platforms will require (Sections 8.4–8.5).

### 8.1 Matching encapsulation strategy to disease mechanism is increasingly important

A major future direction is more rational matching of delivery platforms to disease subtype. AGA and AA are often discussed together because both involve hair loss, but the relevant delivery problems are not the same. AGA may benefit from platforms that improve follicular retention, angiogenesis, anti-inflammatory tone, oxidative-stress control, or long-acting exposure to growth-promoting agents (62, 65, 74). AA is more likely to require local immunomodulation, biomarker-responsive release, or combination strategies that help restore immune privilege while limiting systemic immunosuppression (70). Framed in the encapsulation terms of this review, this means the carrier itself should be chosen to match the dominant biology of the subtype rather than treated as a generic penetration aid. For AGA, that points toward regeneration-oriented encapsulation: codelivery liposomes or ion-doped porous particles that sustain the VEGF- and Wnt-driven angiogenesis and anagen induction discussed above. For AA, it points toward immunomodulation-oriented encapsulation: acid-labile frameworks or responsive carriers that concentrate JAK inhibitors or other immunomodulators where follicular inflammation is greatest.

This logic extends within each disease category as well: not every patient with AGA has the same dominant biology, and not every AA lesion has the same inflammatory state. This makes personalized or mechanism-matched formulation selection a plausible long-term direction, particularly for systems that combine drug release with local sensing or for microneedles carrying multi-agent depots that can be tailored to a scalp phenotype rather than deployed as one-size-fits-all patches.

### 8.2 Manufacturing, quality control, and storage stability remain major bottlenecks

Almost every advanced alopecia platform reviewed here faces chemistry, manufacturing, and controls (CMC) challenges. While many studies demonstrate encouraging biologic activity or improved follicular delivery at laboratory scale, translation requires robust, reproducible, and scalable manufacturing processes that can consistently produce dosage forms with tightly controlled composition, architecture, and performance. This is particularly important for complex carriers such as nanoliposomes, porous scaffolds, hydrogels, and dissolving or coated microneedle systems, where small variations in particle size, porosity, crosslinking density, drug loading, or release kinetics can substantially alter therapeutic outcomes.

These considerations also carry formal regulatory weight. Products that combine a device-like array, a drug, and a nanocarrier are likely to be regulated as combination products. Development will require GMP-compliant manufacturing, an appropriate microbial-control or sterility strategy, and predefined batch-release specifications for attributes such as dose uniformity, needle mechanics, *in vitro* release, vesicle size, and residual reagents. These tests and acceptance criteria should be incorporated early in development.

For microneedle-based systems, manufacturing challenges are especially pronounced because both the formulation and the physical geometry of the array must be tightly controlled. Developers must ensure reliable needle sharpness, mechanical strength, insertion depth, and fracture resistance across large batches, while also maintaining dose uniformity from needle to needle and patch to patch. In the final product, reliable insertion mechanics are essential, as inconsistent penetration into the scalp could lead to underdosing, variable follicular exposure, and reduced efficacy. This issue may be even more significant in alopecia patients with thicker scalp skin, fibrosis, inflammation, or substantial hair coverage, all of which can interfere with patch–skin contact and reproducible application.

In addition to insertion performance, moisture sensitivity remains a major concern for many polymeric and dissolvable systems. Hygroscopic materials may soften, swell, or prematurely dissolve during storage, compromising needle geometry and reducing mechanical integrity before use. Inconsistent drug loading or premature release can blunt the angiogenic, anti-inflammatory, and hair-cycle-modulating signals (VEGF- and Wnt-driven anagen induction, perifollicular angiogenesis, immunomodulation) on which these platforms depend, so a patch that has lost potency in storage may fail to activate follicles even if it inserts correctly. Lipid-based carriers and biologic payloads are especially vulnerable: liposomes, growth factors, cytokines, and JAK-pathway modulators may undergo hydrolysis, oxidation, aggregation, or drug leakage over time, and loss of payload bioactivity can quietly erode the immunomodulatory and regenerative performance of the product. These issues underscore the need for careful excipient selection, residual-moisture control, and protective packaging strategies such as foil pouches, desiccants, inert atmospheres, or cold-chain storage when necessary. However, such measures can increase cost and logistical complexity, which may hinder large-scale deployment or consumer adoption.

Two further points connect these manufacturing realities directly to the clinical promise of encapsulation-integrated platforms for immunomodulation and follicular regeneration. First, the very encapsulation step that gives these systems their biological advantage is also their most fragile manufacturing attribute. For nanoliposome–microneedle products in particular, vesicle integrity must survive casting, drying, and insertion, and processing must reproducibly control encapsulation efficiency, vesicle size, and lamellarity at scale, because drug leakage during fabrication can blur the line between a true carrier-mediated depot and a simple mixed formulation—and with it the mechanistic and regulatory rationale for the product. Encouragingly, immobilizing vesicles within a dry, low-water microneedle matrix can itself reduce the aqueous-phase instability that limits liposome suspensions, so dry-state storage may be part of the solution rather than only a liability. Second, every added layer of complexity (a porous framework, a responsive trigger, a biologic payload, a sensing module) multiplies the number of attributes that must be held constant from batch to batch. For a chronic, quality-of-life condition like alopecia, where patients weigh a sophisticated patch against simple topical MXD or an oral JAK inhibitor, this complexity bears directly on therapeutic reproducibility, cost, and ultimately adoption: a platform whose immunomodulatory or regenerative performance drifts from lot to lot, or that is too costly or cumbersome to apply, will struggle to reach the clinic regardless of how compelling its preclinical biology appears.

A practical scale-up strategy should translate these concerns into a quality-by-design framework that links critical material attributes and process parameters to predefined critical quality attributes. High-throughput molding, carrier production, casting, drying, and packaging processes should be validated with in-line or at-line inspection of needle geometry and defect rates, stability-indicating assays for potency, leakage, and degradation, and statistical controls that demonstrate reproducibility across lots, operators, equipment, and manufacturing sites. Regulatory planning should begin early to clarify the combination-product pathway and the respective drug, device, and biologic requirements, including extractables and leachables, container-closure integrity, transport validation, human-factors testing, and post-market surveillance. Commercial viability will also depend on unit cost, shelf life without an impractical cold chain, reliable application through hair, ease of self-administration, reimbursement, and a clinically meaningful advantage over inexpensive topical or oral therapies.

### 8.3 Long-term local, immune, and follicular-niche effects remain underdefined

The features that make encapsulation systems attractive (prolonged residence, sustained release, and bioactive carriers) are also what make their long-term effects on the scalp hard to predict. Preclinical studies commonly report acceptable short-term cytocompatibility and favorable histology, but alopecia treatment is usually chronic. Repeated application raises questions about barrier recovery, micro-scarring, cumulative inflammation, local immune sensitization, follicular fibrosis, and the tissue fate of any retained nano- or microstructured carrier.

The prolonged local residence and sustained-release profiles that define these platforms have a direct safety corollary: a depot that releases drug for 28 days, or a porous framework that degrades slowly, also keeps its material in contact with perifollicular tissue for that entire window. This matters most for retained inorganic components (zirconium, zinc, iron, or silicon from porous carriers) whose slow clearance could drive chronic low-grade inflammation or immune sensitization. In AA in particular, any persistent pro-inflammatory stimulus risks reawakening the cytotoxic loop that therapy is meant to quiet.

Equally important, and easy to overlook when safety is framed only as toxicity or biocompatibility, are the uncertainties specific to the biological mechanisms these platforms are designed to engage. Chronic local immunomodulation is itself an open question: repeatedly damping IFN-γ/IL-15 signaling or NF-κB activity in the scalp may restore follicular immune privilege, but the long-term consequences of sustained local immune suppression for normal follicular defense and immune surveillance are unknown. The durability of regeneration is similarly unproven: whether follicles driven into anagen by intermittent angiogenic and Wnt-promoting signals remain in a healthy cycling state once treatment stops, and whether years of repeated encapsulated-carrier exposure remodel the follicular niche itself, has not been established. Stimulus-responsive carriers add a further layer: a pH- or ion-triggered system behaves differently in inflamed versus quiescent scalp, so the release kinetics (and the resulting immune and regenerative effects) measured in one disease state may not transfer to another, and locally released metal ions could perturb the same homeostatic signals the therapy aims to normalize. Taken together, these considerations argue for reserving the most complex encapsulation strategies for cases that genuinely require a depot or a responsive trigger; where the goal is simply better short-term penetration of a well-tolerated drug, the added material complexity may not be justified.

Future safety studies should therefore move beyond simple viability assays and short murine treatment windows. Useful next steps would include repeat-dose scalp studies, histologic mapping of perifollicular fibrosis, quantitative biodistribution or clearance studies for retained components, explicit assessment of whether repeated microneedle application and prolonged local release alter normal follicular cycling dynamics over time, and evaluation of these effects in healthy as well as diseased tissue. Without those data, it will remain difficult to argue convincingly that a sophisticated local platform is safer than optimized topical treatment or selective systemic therapy.

### 8.4 Clinical translation is still sparse and will require earlier regulatory planning

Despite rapid preclinical innovation, clinical translation remains limited. Current trial records include adjacent localized-delivery or penetration-enhancement studies, such astopical sildenafil-loaded lipid nanocarries for AA and comparisons of topical MXD NPs after microneedling or fractional-laser treatment (112, 113). They do not yet provide mature clinical evidence for the encapsulation-integrated microneedle platforms reviewed herein. Earlier and broader translational planning is therefore needed.

A credible clinical-development roadmap should define several elements in advance. Primary efficacy endpoints should include trichoscopic hair density and diameter and, where appropriate, the anagen:telogen ratio. Biomarkers such as VEGF, Wnt-pathway activity, CD34, Ki-67, perifollicular CD8^+^NKG2D^+^ infiltrates, and IL-15/IFN-γ should be treated as exploratory mechanistic endpoints unless they are clinically validated. Studies should also benchmark against topical or oral MXD for AGA and approved JAK inhibitors for AA, address patient-use factors, and plan early for release testing, microbial control, human factors, and chronic-use toxicology. The strongest programs will match formulation complexity to a clearly defined clinical problem.

### 8.5 The future of encapsulation-integrated microneedle platforms for alopecia

The defining ambition of the next generation will be to move past drug delivery as an end in itself: future platforms should aim not only to deliver a drug efficiently but to actively regulate the follicular immune microenvironment and promote sustained hair regeneration. We believe the most promising of these platforms will therefore be mechanism-matched, structurally modular, and designed with manufacturability in mind. Just as importantly, the next generation should treat regenerative biology and immunomodulation as central design principles rather than downstream consequences of delivery. Future platforms should actively support follicular regeneration, immune regulation, and microenvironment remodeling by carrying biologic payloads, using bioinstructive materials, and tailoring the immunological payload to the specific disease and lesion state.

Emerging cargo classes could extend these platforms beyond conventional small molecules. Biologics, including cytokines, antibodies, growth factors, and peptides, could be localized to restore follicular immune privilege or stimulate regeneration while limiting systemic exposure, but they require protection against denaturation and proteolysis. Nucleic acid approaches, such as small interfering RNA, microRNA, messenger RNA, and viral or nonviral gene delivery, could silence androgen or inflammatory pathways or sustain expression of regenerative factors; cell-specific uptake, innate immune activation, reversibility, and dose control will be central safety considerations. A recent study bioorthogonally immobilized adeno-associated virus encoding acidic fibroblast growth factor on a microneedle interface, providing localized, sustained transgene expression and improved hair regeneration in murine alopecia models (114). Exosomes and other extracellular vesicles offer naturally packaged proteins, lipids, and regulatory RNAs, and sustained microneedle-mediated delivery of stem-cell-derived exosomes has already promoted Wnt-associated hair regeneration in preclinical models (115). However, source heterogeneity, purification, potency assays, cargo variability, storage, and regulatory classification remain substantial barriers. Combination microneedles may pair an immunomodulator with an angiogenic or regenerative cue, or program sequential release, but synergy should be demonstrated against each monotherapy and balanced against the greater formulation, interaction, and quality-control burden of a fixed combination (114, 115).

One hypothesis for future study is that fragmented nanofibers could be integrated into or delivered alongside MN arrays to improve local retention or provide extracellular-matrix-like cues. Collagen-based fibers are a plausible candidate, but direct evidence for improved follicular regeneration in alopecia models is currently lacking.

More broadly, the field should continue shifting from “microneedles as penetration enhancers” to “microneedles as integrated follicular-intervention systems.” That means designing platforms capable of controlling drug localization, residence time, immune state, angiogenic tone, and possibly real-time tissue readouts. If future studies can pair that systems-level ambition with stronger translational rigor, these systems have real promise as therapeutic options for patients with chronic hair loss.

## 9 Conclusion

Encapsulation-integrated microneedles can convert a brief penetration event into localized cargo protection, controlled release, co-delivery, or stimulus-responsive therapy. Across the platforms reviewed, nanoparticles and nanoliposomes improve cargo stability and targeting, porous carriers provide high loading and tunable depots, microspheres enable prolonged biodegradable release, and light-guiding arrays extend microneedles beyond drug delivery. These functions have been associated with reduced inflammatory signaling, improved angiogenesis, activation of Wnt/beta-catenin and other regenerative pathways, and transition of follicles into anagen. However, the evidence remains predominantly preclinical, release and outcome methods are heterogeneous, head-to-head comparisons are scarce, and long-term pharmacokinetics, carrier clearance, repeat-dose scalp safety, and clinical benefit over established treatments remain insufficiently defined.

Next-generation systems should therefore be mechanism-matched, manufacturable, and clinically testable rather than complex for their own sake. Priorities include reproducible GMP production and standardized quality controls; chronic-use safety and biodistribution studies; comparator-controlled trials with clinically meaningful hair and patient-reported outcomes; and rational development of biologics, nucleic acids, extracellular vesicles, and combination therapies. Smart or responsive materials should be advanced only when their trigger, added benefit, and commercial feasibility are demonstrated. Meeting these requirements could position microneedle-based therapies as practical, personalized, and locally acting options for alopecia while minimizing systemic exposure and treatment burden.

## Glossary

AA: Alopecia Areata
AGA: Androgenetic Alopecia
Akt: Protein Kinase
ATP: Adenosine Triphosphate
BSP: Bletilla striata Polysaccharide
BSP: Bella Striata Polysaccharide
CAP: Cold Atmospheric Plasma
CCL22: CC-Motif Chemokine Ligand 22
CD3: Cluster of Differentiation 3
CD31: Cluster of Differentiation 31 (PECAM-1; endothelial/microvascular marker)
CD34: Cluster of Differentiation 34 (follicular stem-cell and neovascular marker)
CD4⁺: Cluster of differentiation 4-positive T lymphocyte
CD44: Cluster of Differentiation 44
CD8+NKG2D+ T cells: Cytotoxic CD8-positive T cells expressing the NKG2D activating receptor
CLSM: Confocal Laser Scanning Microscopy
CMC: Chemistry, Manufacturing, and Controls
CXCL1: C-X-C Motif Chemokine Ligand 1
CXCL10: C-X-C Motif Chemokine Ligand 10
CXCL11: C-X-C Motif Chemokine Ligand 11
CXCL9: C-X-C Motif Chemokine Ligand 9
DHT: Dihydrotestosterone
DUT: Dutasteride
ECM: Extracellular Matrix
FDA: Food and Drug Administration
GA: Glycyrrhizic Acid
GA@ZIF-8: Glycyrrhizic Acid Adsorbed into ZIF-8 (“licorzinc” MOF)
GMP: Good Manufacturing Practice
H&E: Hematoxylin and Eosin
HA: Hyaluronic Acid
HDP: Hair Dermal Papilla
HF: Hair Follicle
HUVEC: Human Umbilical Vein Endothelial Cell, IFN-γ, Interferon Gamma
IL-15: Interleukin-15
IL-1β: Interleukin-1 Beta
IL-2/IL-15: Interleukin-2/Interleukin-15
IκBα: Inhibitor of Nuclear Factor Kappa-B Alpha
JAK: Janus Kinase
Ki67: Marker of Cellular Proliferation Ki67
Ki67: Marker of Nuclear Protein Associated with Cellular Proliferation
Ki-67: Proliferation Marker
KK-NLPs: Kopexil and Kopyrrol Co-encapsulated Nanoliposomes
LED: Light-Emitting Diode
LLLT: Low-Level Light Therapy
LMNP: Light-Guiding Microneedle Patch
MAPK: Mitogen-activated Protein Kinase
MN(s): Microneedle(s)
MOF: Metal-organic Framework
MS(s): Microsphere(s)
MXD: Minoxidil
NF-κB: Nuclear Factor Kappa B
NF-κB: Nuclear Factor kappa-light-chain-enhancer of activated B cells
NKG2D: Natural-killer group 2D (activating immune receptor)
NLC: Nanostructured Lipid Carrier
NLP(s): Nanoliposome(s)
NLRP3: NLR Family Pyrin Domain Containing 3
NP(s): Nanoparticle(s)’
PCL: Poly(ϵ-caprolactone)
PHA: Polyhydroxyalkanoate
PLA: Polylactic Acid
PLGA: Poly(lactic-co-glycolic acid)
PMMA: Polymethyl Methacrylate
PQFN: Ferrum-chelated Puerarin/Quercetin Nanoparticles
PVA: Polyvinyl Alcohol
PVP: Polyvinylpyrrolidone
QFN: Quercetin Nanoparticles
qRT-PCR: Quantitative Reverse Transcription Polymerase Chain Reaction
Qu: Quercetin
Rg3: Ginsenoside Rg3
Rg3-LPs/Rg3-MNs: Ginsenoside Rg3 liposomes/Rg3-liposome-loaded microneedles
ROS: Reactive Oxygen Species
SALT: Severity of Alopecia Tool
SD: Standard Deviation
SRD5A2: Steroid 5 alpha-reductase 2 gene
TA: Triamcinolone Acetonide
TCD(s): Triamcinolone Candlelit Dissolving Microneedle(s)
TE: Telogen Effluvium
TFB: Tofacitinib
TFB@NP-MNs: Tofacitinib-loaded Nanoparticle-integrated microneedles
TGF-β1: Transforming Growth Factor Beta 1
TPy: Terpyridine
Treg: Regulatory T cell
UiO-66: University of Oslo-66 (zirconium-based metal-organic framework)
VEGF: Vascular Endothelial Growth Factor
Wnt3a/Wnt10b: Wnt Family Signaling Ligands 3a and 10b
ZCQ: Zinc/copper dual-doped Mesoporous Silica Loaded with Quercetin
ZCQ/MN: ZCQ Nanocomposite Microneedle Patch
ZIF-8: Zeolitic Imidazolate Framework-8 (zinc-based)
